# A Screen for Modifiers of Cilia Phenotypes Reveals Novel MKS Alleles and Uncovers a Specific Genetic Interaction between *osm-3* and *nphp-4*

**DOI:** 10.1371/journal.pgen.1005841

**Published:** 2016-02-10

**Authors:** Svetlana V. Masyukova, Dawn E. Landis, Scott J. Henke, Corey L. Williams, Jay N. Pieczynski, Kelly N. Roszczynialski, Jannese E. Covington, Erik B. Malarkey, Bradley K. Yoder

**Affiliations:** 1 Department of Cell, Developmental, and Integrative Biology, University of Alabama at Birmingham Medical School, Birmingham, Alabama, United States of America; 2 Department of Pharmacology and Therapeutics, College of Medicine, University of Florida, Gainesville, Florida, United States of America; Seattle Children's Research Institute, UNITED STATES

## Abstract

Nephronophthisis (NPHP) is a ciliopathy in which genetic modifiers may underlie the variable penetrance of clinical features. To identify modifiers, a screen was conducted on *C*. *elegans nphp-4(tm925)* mutants. Mutations in ten loci exacerbating *nphp-4(tm925)* ciliary defects were obtained. Four loci have been identified, three of which are established ciliopathy genes *mks-1*, *mks-2*, and *mks-5*. The fourth allele (*yhw66*) is a missense mutation (S316F) in OSM-3, a kinesin required for cilia distal segment assembly. While *osm-3(yhw66)* mutants alone have no overt cilia phenotype, *nphp-4(tm925);osm-3(yhw66)* double mutants lack distal segments and are dye-filling (Dyf) and osmotic avoidance (Osm) defective, similar to *osm-3(mn357)* null mutants. In *osm-3(yhw66)* mutants anterograde intraflagellar transport (IFT) velocity is reduced. Furthermore, expression of OSM-3(S316F)::GFP reduced IFT velocities in *nphp-4(tm925)* mutants, but not in wild type animals. *In silico* analysis indicates the S316F mutation may affect a phosphorylation site. Putative *phospho-null* OSM-3(S316F) and *phospho-mimetic* OSM-3(S316D) proteins accumulate at the cilia base and tip respectively. FRAP analysis indicates that the cilia entry rate of OSM-3(S316F) is slower than OSM-3 and that in the presence of OSM-3(S316F), OSM-3 and OSM-3(S316D) rates decrease. In the presence OSM-3::GFP or OSM-3(S316D)::GFP, OSM-3(S316F)::tdTomato redistributes along the cilium and accumulates in the cilia tip. OSM-3(S316F) and OSM-3(S316D) are functional as they restore cilia distal segment formation in *osm-3(mn357)* null mutants; however, only OSM-3(S316F) rescues the *osm-3(mn357)* null Dyf phenotype. Despite rescue of cilia length in *osm-3(mn357)* null mutants, neither OSM-3(S316F) nor OSM-3(S316D) restores ciliary defects in *nphp-4(tm925);osm-3(yhw66)* double mutants. Thus, these OSM-3 mutations cause NPHP-4 dependent and independent phenotypes. These data indicate that in addition to regulating cilia protein entry or exit, NPHP-4 influences localization and function of a distal ciliary kinesin. Moreover, data suggest human OSM-3 homolog (Kif17) could act as a modifying locus affecting disease penetrance or expressivity in NPHP patients.

## Author Summary

Nephronophthisis (NPHP) is a genetically heterogeneous ciliopathy that has minimal genotype-phenotype correlation. The cause of this variation is not known, but could result from additional mutations in the patients’ backgrounds capable of modifying the phenotype. To identify candidate NPHP modifying loci, we conducted an enhancer mutagenesis screen using *C*. *elegans nphp-4(tm925)* mutants. Mutations in ten loci were obtained that severely exacerbated the cilia defects in the *nphp-4(tm925)* mutants, but importantly, had minimal defects in the absence of the *nphp-4* mutation. Here we identified four of these loci, each encoding a cilia protein. Three mutations are in known ciliopathy genes, *mks-1*, *mks-2* and *mks-5*. The fourth allele is a missense (S316F) mutation in OSM-3, a kinesin required for distal cilia assembly and is the sole kinesin responsible for intraflagellar transport along the cilia distal segment in *C*. *elegans*. The *osm-3(yhw66)* mutation affects a putative phosphorylation site that is important for OSM-3 localization, movement, and function, largely in an *nphp-4* dependent manner. These data establish a genetic interaction between *osm-3* and *nphp-4* that regulates kinesin activity and localization and raises the possibility that mutations in *Kif17*, the mammalian homolog of *osm-3*, may influence the phenotypes in human NPHP patients.

## Introduction

Defects in cilia assembly or function underlie a large group of developmental disorders and disease syndromes collectively called the *ciliopathies* [1–3]. Nephronophthisis (NPHP) is a genetically heterogeneous ciliopathy with cysts forming at the corticomedullary junction in the kidney [4]. Intriguingly, mutations in several NPHP genes have been identified as the cause of several other ciliopathies that have some overlapping yet clinically very distinct phenotypes. For example, mutations in RPGRIP1L (NPHP8/MKS5) can cause an NPHP phenotype, but also Meckel-Grüber syndrome (MKS), a pre- or perinatal lethal condition due to neural tube closure and heart defects, or Joubert syndrome (JBTS) that has cystic kidneys, mental retardation, cerebellar hypoplasia, ataxia, and developmental abnormalities [5, 6]. Additionally, patients with identical mutations in NPHP4 can present with typical NPHP but also with retinitis pigmentosa when classified as Senior-Löken Syndrome (SLS) [7–11]. The distinct phenotypes observed in NPHP, MKS, JBTS, and SLS patients along with the genetic overlap suggest that there are epistatic background mutations functioning to exacerbate the cilia related phenotypes.

In previous studies, we determined that similar genetic effects are observed in *Caenorhabditis elegans* with *nphp* and *mks* gene mutations. Individual *nphp* or *mks* mutants have mild defects in cilia structure and function [12–16]. In contrast, *C*. *elegans* with mutations in both an *nphp* and *mks* gene have severe ciliary defects that include malformed, misoriented, mispositioned, and loss of cilia. Further, we demonstrated that several proteins involved in NPHP and MKS form two distinct complexes that both localize to the base of cilia in a region called the transition zone (TZ) [12, 13, 17]. Both complexes are required for proper function of the TZ as a ciliary gate to regulate what proteins are present in the cilium and thus establish the cilium as a distinct compartment from the rest of the cell. Collectively these data indicate that there are strong functional interactions between proteins involved in MKS and NPHP and that mutations in these genes are likely candidates for modifying NPHP and other ciliopathy phenotypes.

*C*. *elegans* is a powerful model to assess genetic interactions between ciliary proteins since unlike in mammalian systems, cilia are not required for normal development or viability. In addition, cilia function and morphological defects can easily be assessed in *C*. *elegans* where mutants disrupting cilia structure are unable to uptake lipophilic dye through the cilia (dye filling defective, Dyf) and cilia morphology can be visualized by expression of a fluorescent tagged ciliary protein. In addition, cilia defects that cause chemotaxis (Che) and osmotic avoidance (Osm) abnormalities are easily analyzed through multiple functional tests [18, 19].

To identify potential NPHP modifier loci we utilized *C*. *elegans nphp-4(tm925)* null mutants as the initiating point for an EMS enhancer screen. From this screen we isolated ten independent strains that showed more severe ciliary defects than observed in *nphp-4* mutants alone and were also dependent upon having the *nphp-4(tm925)* mutation in the background. Here we report the identification of four of the mutations as new alleles affecting MKS proteins as well as OSM-3, a kinesin required for assembly of the distal segment of cilia.

## Results

### The *nphp-4* mutant synthetic Dyf screen

**Fig 1:**
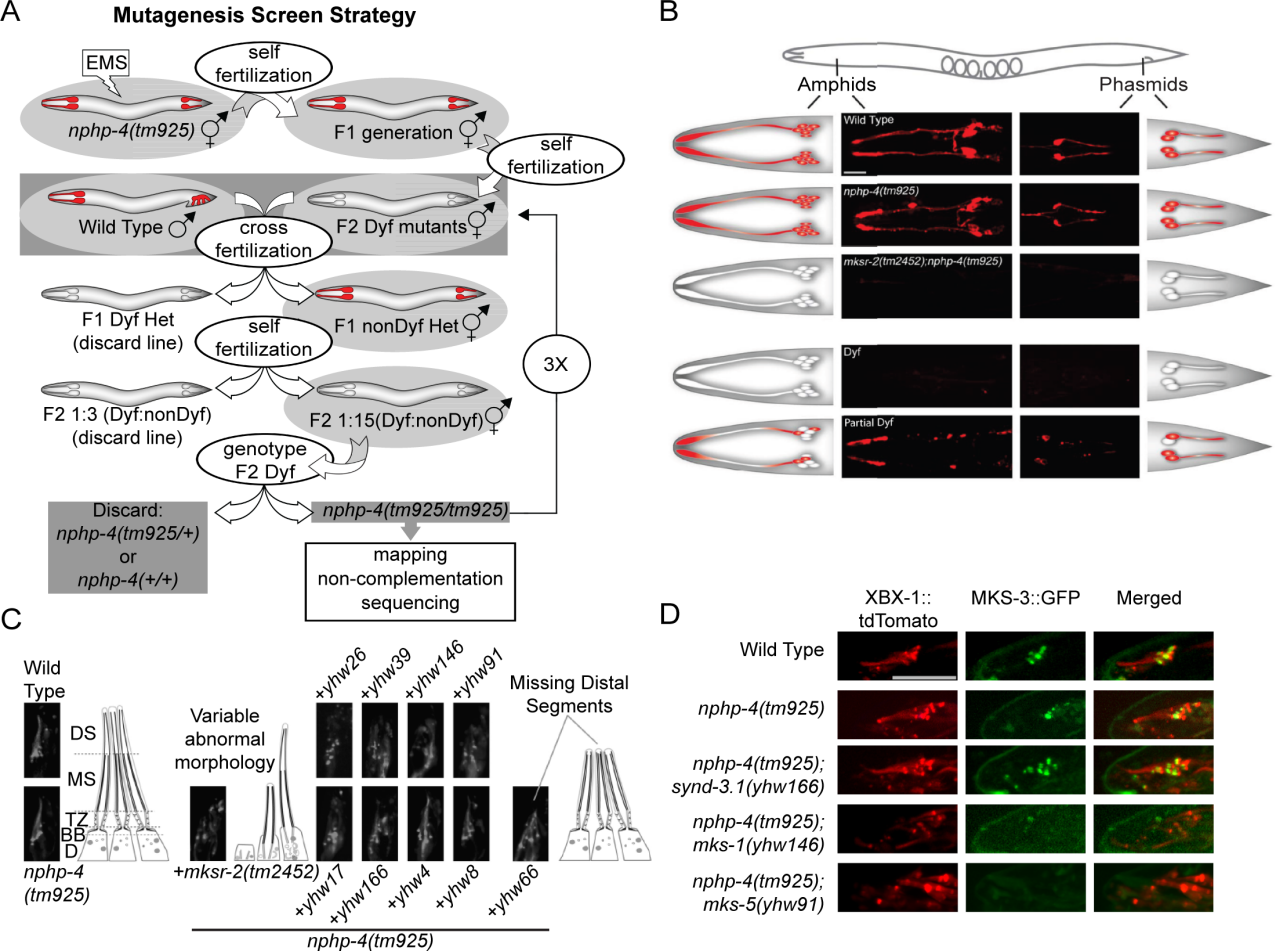
*Nphp-4* synDyf enhancer mutagenesis screen. (A) Illustration depicting the screening strategy used to generate and select for the mutants used in this paper. Grey shading indicates worms that were maintained for the next step of the screen. (B) Representative confocal images and illustrations of amphid (head) and phasmid (tail) neurons of dye-filled animals depicting how worms obtained in the screen were classified into Dyf and partial Dyf phenotypes. Scale bar is 21 μm. (C) Representative confocal images of amphid cilia of mutants obtained in the screen using the cilia axonemal marker XBX-1::TdTomato to show cilia morphology. Corresponding illustrations are shown, modified with permission from Williams et al., 2011 [25]. D, distal dendrite; BB, basal body; TZ, transition zone; MS, middle ciliary axoneme segment; DS, distal ciliary axoneme segment. (D) Confocal images of amphid cilia co-expressing the cilia marker XBX-1::tdTomato and TZ MKS module protein MKS-3::GFP to analyze MKS-3 localization in the compound mutant backgrounds. Scale, 9μm. For full MKS-3 localization dataset in other mutant strains, see Table 1.

To uncover possible NPHP modifier loci, we mutagenized *nphp-4(tm925*) null mutant worms with EMS and screened 4,000 haploid genomes and 500,000 individual F2 worms for dye-filling defects (Dyf) (Fig 1A). We isolated 118 lines that maintained a consistent transmittable Dyf phenotype. These 118 mutant lines were outcrossed for at least three generations to wild-type worms to reduce the amount of background mutations. After each outcrossing, lines whose progeny had a 1:3 Dyf to wild-type segregation ratio, indicative of homozygous recessive mutations at a single locus, (e.g. intraflagellar transport (IFT) mutations that would be Dyf independent of the *nphp-4* mutation) were eliminated from the study. Lines showing a significant deviation from the 1:3 Dyf to wild-type segregation ratio (e.g. 1:15), indicative of double homozygous recessive inheritance at two unlinked loci, were selected for further analysis. To confirm that the Dyf phenotype was dependent on the *nphp-4(tm925)* mutation, Dyf and non-Dyf progeny at each round of outcrossing were genotyped for the homozygous *nphp-4* deletion. All Dyf worms were homozygous mutant for *nphp-4*. After outcrossing, 29 double homozygous recessive *nphp-4;synd-x* (*synthetic dyf* genes where x represents the unknown gene) Dyf lines were established. Lines were further categorized based on the severity of their Dyf phenotype, which we assigned as being either Dyf or partial Dyf (Fig 1B and Table 1). A Dyf phenotype was defined as being equivalent to what is normally observed in an IFT null mutant and partial Dyf was defined as those that retain some neuronal fluorescence different than what is seen in *nphp-4(tm925)* mutants alone (Fig 1B).

**Table 1:** *nphp-4* synthetic Dyf screen alleles and phenotypes.

Gene	Linkage Group	Strain	Gene Identity	Dye Filling	MKS-3::GFP
*synd-x2*.*1*	II	*YHW34*		Dyf	-
* *		*YHW 35*		Dyf	-
* *		*YHW 36*		Dyf	missing from TZ
* *		*YHW 39*		Dyf	missing from TZ
* *		*YHW 128*	*mks-2*	Dyf	missing from TZ
* *		*YHW 129*		Dyf	-
*synd-x2*.*2*	II	*YHW 91*	*mks-5*	partial Dyf	missing from TZ
*synd-x3*.*1*	III	*YHW 62*		partial Dyf	at TZ
* *		*YHW 166*		partial Dyf	at TZ
*synd-x3*.*2*	III	*YHW 146*	*mks-1*	Dyf	missing from TZ
*synd-x4*.*1*	IV	*YHW 4*		partial Dyf	at TZ
*synd-x4*.*2*	IV	*YHW 66*	*osm-3*	Dyf	at TZ
*synd-x5*.*1*	V	*YHW 3*		partial Dyf	at TZ
* *		*YHW 5*		partial Dyf	-
* *		*YHW 9*		partial Dyf	-
* *		*YHW 10*		partial Dyf	-
* *		*YHW 12*		Dyf	at TZ
* *		*YHW 14*		partial Dyf	-
* *		*YHW 15*		partial Dyf	-
* *		*YHW 17*		Dyf	at TZ
* *		*YHW 19*		Dyf	at TZ
* *		*YHW 130*		Dyf	at TZ
* *		*YHW 131*		Dyf	-
* *		*YHW 153*		Dyf	-
*synd-x5*.*2*	V	*YHW 6*		partial Dyf	at TZ
* *		*YHW 133*		partial Dyf	-
*synd-x6*.*1*	X	*YHW 24*		partial Dyf	-
* *		*YHW 26*		partial Dyf	at TZ
*synd-x6*.*2*	X	*YHW 71*		partial Dyf	at TZ

Summary of the screen results. Shown is gene/locus designation, chromosomal location of alleles, and associated phenotypes.

### Ciliary defects in *nphp-4; synd-x* double mutants

Using a dynein light intermediate chain XBX-1::tdTomato to mark ciliary axonemes [20, 21], we assessed cilia morphology in *nphp-4;synd-x* double mutants generated from the screen (Fig 1C). Most *nphp-4;synd-x* strains exhibited varying degrees of cilia structure, bundling, and positioning defects (Fig 1C and S1 Fig). For example, cilia appear to be nearly absent in the *yhw26*, truncated in *yhw66*, and nonfasiculated and misoriented in *yhw39*; but were minimally altered in *yhw166*, *yhw4*, and *yhw8* (Fig 1C).

Since previous data indicated there is a genetic interaction between mutations in *nphp-4* and several *mks* genes that can cause a synthetic Dyf phenotype, we assessed whether any of the new mutations affect the formation of the MKS complex within the TZ [12–15]. This was done by analyzing MKS-3::GFP localization in each of the strains (Table 1 and Fig 1D). MKS-3 is one of the most peripheral MKS proteins in the MKS complex and is frequently lost from the TZ in other *mks* mutant backgrounds [13]. While seven loci had no overt effect on MKS-3::GFP TZ localization, in three mutant lines MKS-3::GFP was not properly localized (Table 1 and Fig 1D) indicating they do disrupt MKS complex formation.

### *nphp-4;synd-x* complementation groups

Single nucleotide polymorphism (SNP) mapping analysis was completed according to Davis et al. (2005) [22, 23] to assign the mutations to a linkage group (Table 1). To establish the number of loci generated from the enhancer screen, we performed non-complementation analysis between each isolated line whose mutations were mapped to the same chromosome. These 29 strains were found to group into ten independent loci (Table 1), although it was difficult to evaluate some loci on linkage group V due the proximity of the *nphp-4* mutation.

To determine whether any of the isolated lines had mutations in known *mks* genes, we performed non-complementation analysis by crossing them with previously known *mks* mutants located on the same linkage group (Table 1). We found that three mutant alleles (*yhw146*, *yhw128*, and *yhw91*) failed to complement the Dyf phenotypes of *nphp-4(tm925);mks-1(tm2705)*, *nphp-4;mks-2(mx1198)*, *and nphp-4;mks-5(tm3100)* animals respectively, indicating that these alleles are likely mutations in *mks-1*, *mks-2*, and *mks-5*. These are also the lines that disrupted MKS-3::GFP localization in the TZ.

### Analysis of novel mks alleles

#### *yhw146* is a novel allele of *mks-1*

**Fig 2:**
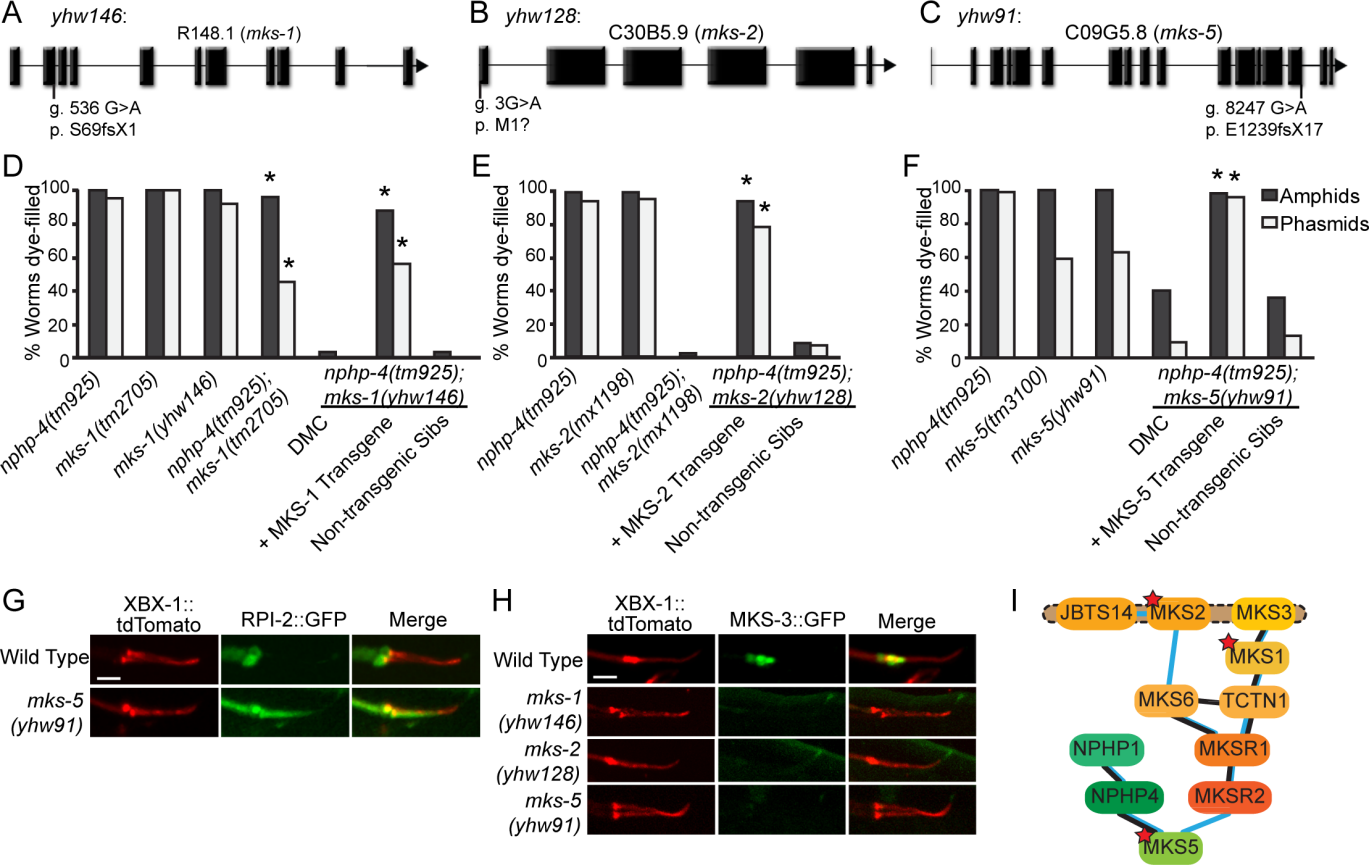
Identification of novel *mks-1*, *mks-2*, *and mks-5* alleles which genetically interact with *nphp-4(tm925)*. (A) Schematic depicting the genomic location of a splice site mutation in exon 2 of *mks-1* found in YHW146. (B) Schematic depicting genomic location of a mutation in the start codon of *mks-2* found in YHW128. (C) Schematic depicting genomic location of the splice site mutation in exon 15 of *mks-5* found in YHW91. (D-F) Dye-filling analysis of the indicated strains demonstrating that the synthetic Dyf (synDyf) phenotype requires mutations in *nphp-4* along with the EMS induced mutation in (D) *mks-1(yhw146)*, (E) *mks-2(yhw128)*, and (F) *mks-5(yhw91)*. The restoration of dye filling in the double mutants expressing the corresponding wild type *mks* gene from an extrachromosomal array confirms that mutations in the *mks* genes is causing the synDyf phenotype when in combination with the *nphp-4(tm925)* allele. DMC, double mutant control. N = 3 trials with at least 100 animals per trial. * = p<0.05 as compared to DMC. (G) Confocal images of the phasmid cilia of wild-type and *mks-5(yhw91)* mutants co-expressing RPI-2::GFP and XBX-1::tdTomato. RPI-2::GFP is able to aberrantly enter the cilium of the *mks-5(yw91)* mutants, but not wild type controls. Scale bar 2μm. (H) Confocal images of wild-type, *mks-1(yhw146)*, *mks-2(yhw128)*, and *mks-5(yhw91)* animals co-expressing MKS-3::GFP and XBX-1::tdTomato in phasmid cilia. MKS-3::GFP fails to localize in the TZ of *mks-1(yhw146)*, *mks-2(yhw128)*, and *mks-5(yhw91)* mutants. Scale bar 2μm. (I) Schematic showing the revised hierarchy map of MKS and NPHP protein assembly in the TZ. Stars indicate MKS proteins with alleles obtained in the screen.

Sequencing the *mks-1* genomic region in *yhw146* mutants revealed a guanine to adenine mutation at the splice donor site of exon 2 (Fig 2A). RT-PCR analysis and cDNA sequence indicate that this mutation impairs splicing extending the reading frame into intron 3 and causing a stop codon that would truncate the protein (S2A Fig). Transgenic rescue analysis confirms the mks-1 mutation as causative in *nphp-4(tm925);mks-1(yhw146)* double mutants (Fig 2D).

The *nphp-4(tm925);mks-1(yhw146)* mutant obtained from our screen is completely Dyf, which is in contrast to the *nphp-4(tm925);mks-1(tm2705)* mutant that was previously shown to be partially dye-filling defective [12]. Thus, the new *mks-1(yhw146)* allele from the screen is a more functionally perturbing mutation than the existing *mks-1(tm2705)* allele. This is in agreement with the original *mks-1(tm2705)* allele, which causes a deletion of 72 amino acids of the third and fourth exon while leaving the reading frame intact, while the *mks-1(yhw91)* allele obtained in the screen is a splice site mutation in exon 2 and would delete the remainder of the protein.

### *yhw128* is a novel allele of *mks-2*

Sequencing of the *mks-2* genomic region of *yhw128* worms revealed a single nucleotide change from guanine to adenine in the start codon (Fig 2B). The next ATG in the sequence would result in an alternate reading frame for MKS-2. Transgenic rescue and non-complementation analyses confirm that the Dyf phenotype in *yhw128* is the result of a novel mutation in *mks-2* (Fig 2E).

### *yhw91* is a novel allele of *mks-5*

Sequencing of the *mks-5* genomic region from the *yhw91* worms revealed a single nucleotide change from guanine to adenine at the splice donor site of exon 15 (Fig 2C). RT-PCR analysis and cDNA sequencing indicates that this mutation disrupts normal splicing of *mks-5* (S2B Fig). It results in a reading frame that extends into intron 15 giving a transcript predicted to encode 17 new amino acids before reaching a stop codon (S2C Fig). Transgenic rescue of the *nphp-4(tm925);mks-5(yhw91)* Dyf phenotype was accomplished with a wild-type copy of MKS-5, confirming the *mks-5* mutation as causative in *yhw91* (Fig 2F).

### Analysis of *mks-1(yhw146)*, *mks-2(yhw128)*, and *mks-5(yhw91)* alleles

We crossed the *mks-1(yhw146)*, *mks-2(yhw128)*, and *mks-5(yhw91)* alleles off of the *nphp-4(tm925)* mutant background to analyze the individual mutation’s effect in the absence of the *nphp-4(tm925)* allele. We first compared their dye-filling capabilities to the previously characterized deletion alleles, *mks-1(tm2705)*, *mks-2(nx111)*, and *mks-5(tm3100)*, respectively [14, 24–27]. Similar to *mks-1(tm2705) and mks-2(nx111)* single mutants, there was no overt defect in dye uptake in *mks-1(yhw146)* or *mks-2(yhw128)* mutants (Fig 2D and 2E). Recent studies of *mks-5(tm3100)* mutants revealed a partially penetrant Dyf phenotype specifically in phasmid (tail) neurons [25, 26]. Identical results were obtained for the *mks-5(yhw91)* mutants (Fig 2F). The Dyf phenotype in *mks-5(yhw91)* mutants was much less severe than observed in the *nphp-4(tm925);mks-5(yhw91)* double mutants and affects both amphid and phasmid neurons.

NPHP and MKS proteins function as part of a TZ barrier controlling protein access to the cilium [25]. This is supported by data showing that non-ciliary membrane-associated proteins, such as RPI-2 and TRAM-1, ectopically enter the cilium in many of the *mks* mutants, including *mks-5(tm3100)* [25]. Similarly, in *mks-5(yhw91)* single mutants there is an abnormal accumulation of RPI-2 within the cilium (Fig 2G) indicating the TZ barrier has been compromised and a functional importance for the lost C-terminal domain of MKS-5 in our *yhw91* mutant.

To further assess whether the mislocalization of MKS-3::GFP in *nphp-4(tm925);mks-1(yhw146)*, *nphp-4(tm926);mks-2(yhw128)*, and *nphp-4(tm925);mks-5(yhw91)* was caused by the genetic interaction with the *nphp-4* mutation, we analyzed MKS-3::GFP localization in *mks-1(yhw146)*, *mks-2(yhw128)*, and *mks-5(yhw91)* single mutants. Previously, we found that MKS-3::GFP localization was unaffected in the *mks-1(tm2705)* background [13]. In direct contrast to these data, MKS-3::GFP was absent from the TZ in the *mks-1(yhw146)* mutants obtained from the screen, again indicating that the new *yhw146* allele is more deleterious than the previously characterized *mks-1*(*tm2705)* mutation (Fig 2H). Importantly, the analysis of this new *mks-1* allele showing mislocalization of MKS-3::GFP allows us to modify the current hierarchy of interactions within the MKS complex and position MKS-1 more proximal to MKS-5 than MKS-3 (Fig 2I) [25]. MKS-3::GFP was also mislocalized in *mks-2(yhw128)* and *mks-5(yhw91)* similarly to the *mks-2(nx111)* and *mks-5(tm3100)* mutants indicating, in contrast to the Dyf phenotype, the assembly of these proteins into their complexes is not dependent on the *nphp-4* mutation (Fig 2H)[12–14, 27]. The identification of several new alleles of *mks* genes that cause a greatly exacerbated cilia phenotype in the presence of an *nphp-4* mutation provides strong justification for this mutagenesis approach to identify modifiers of the NPHP and other ciliopathy phenotypes in *C*. *elegans*.

### A novel *osm-3* allele produces a synthetic Dyf phenotype in the background of *nphp-4(tm925)*

**Fig 3:**
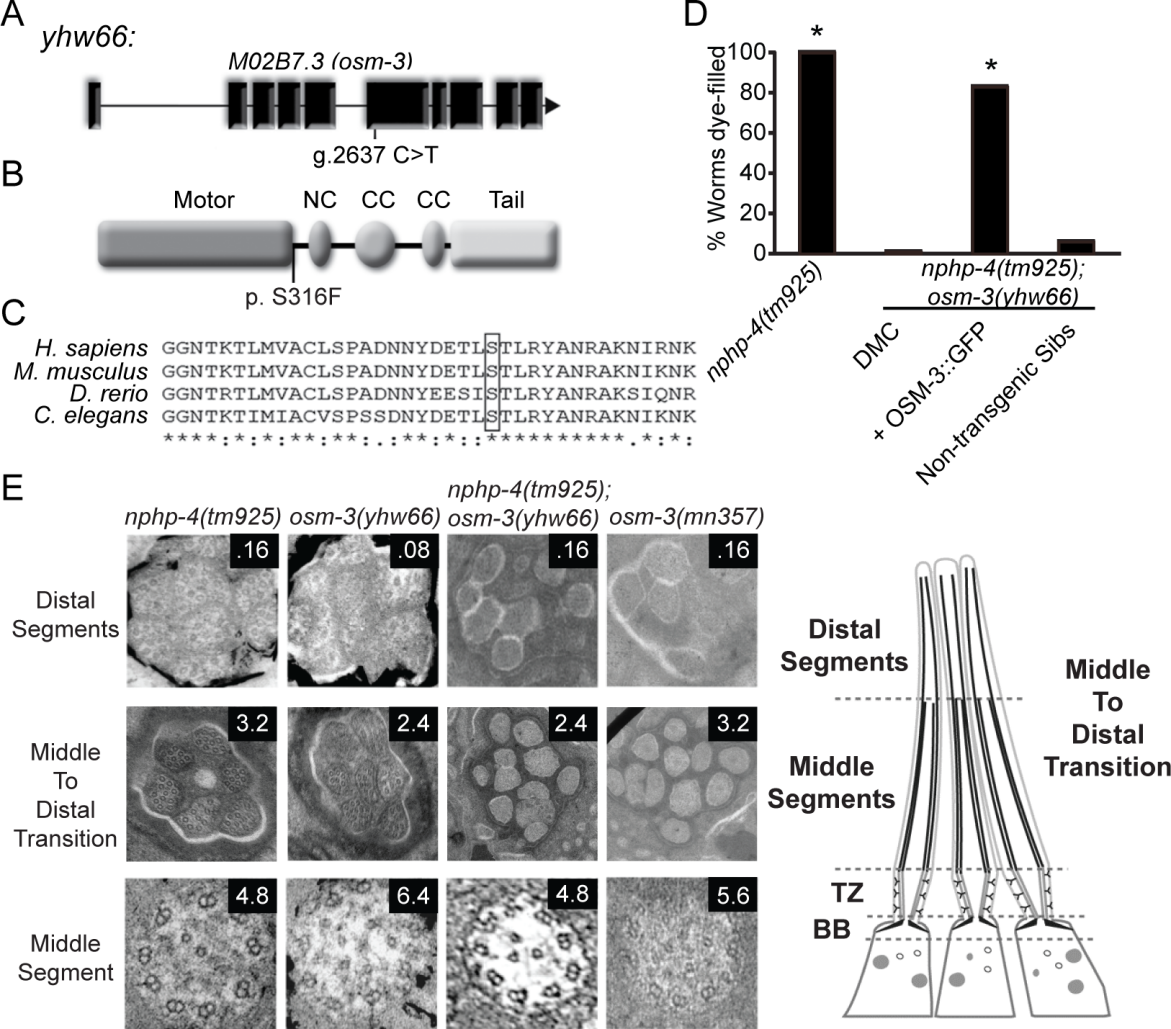
The novel *osm-3(yhw66)* genetically interacts with *nphp-4(tm925)* causing ciliary defects. (A) Schematic of the *osm-3*(*yhw66)* gene showing the C/T substitution at genomic position 2637. (B) Schematic of the OSM-3 protein depicting the S>F amino acid substitution at amino acid positon 316 in YHW66. NC, neck domain; CC coiled-coil motif. (C) Alignment of OSM-3 homologs shows high degree of conservation in the region where *yhw66* mutation is located. (D) Dye-filling analysis of amphid neurons in *nphp-4(tm925)*, *nphp-4(tm925);osm-3(yhw66)*, *nphp-4(tm925);osm-3(yhw66)* expressing OSM-3::GFP transgenic, and nontransgenic siblings confirm that the mutation in *osm-3(yhw66)* is causing the Dyf phenotype in association with the *nphp-4(tm925)* allele. DMC, double mutant control. N = 3 trials with at least 100 animals per trial. * = p < .05 as compared to DMC. (E) Transmission electron micrographs showing cross sections of serial sectioned amphid cilia in the distal segment, middle to distal transition, and middle segment of cilia. Sections are 80nm thick. Numbers refer to distance in μm from distal tip of cilia. TZ, transition zone. BB, basal body.

Through reiterative SNP mapping and whole genome sequencing we identified a missense mutation in the *osm-3* gene in *yhw66* (Fig 3A–3C). OSM-3 is a homodimeric IFT kinesin motor. IFT is a highly conserved process required for cilia assembly that utilizes anterograde-directed kinesin and retrograde-directed dynein motor proteins for movement of proteins along the microtubule axoneme between the base and tip of the cilium. In *C*. *elegans* there are two IFT kinesin complexes in cilia with Osm-3 being the only kinesin that functions on microtubule singlets of the cilium’s distal segment [28–31]. As a consequence, in *osm-3(mn357)* null mutants the proximal region of cilia form while the distal segments do not, resulting in a Dyf phenotype [28, 32, 33]. In the *nphp-4(tm925);osm-3(yhw66*) mutants obtained from the screen the distal segments of cilia appear to be absent and they have a severe Dyf phenotype (Figs 1C and 3D and 3E) closely resembling *osm-3(mn357)* null mutants.

The *osm-3(yhw66*) mutation is a substitution of cytosine for thymine at genomic position 2,637 in exon 6. This results in a serine to phenylalanine substitution at amino acid position 316 (S316F) that is located just within or just downstream of the motor domain (Fig 3A and 3B). A comparison of the primary amino acid sequence across multiple species shows that S316 is highly conserved in other OSM-3 homologs (Fig 3C) and falls within an overall conserved domain found across many different kinesin family members. The specific S316F substitution is not listed in the human SNP database (dbSNP version 132), and the Screening for Non-acceptable Polymorphisms program (SNAP) (http://www.rostlab.org/services/SNAP) predicts that the S316F substitution is non-neutral with 78% accuracy. To confirm that the *osm-3(yhw66)* missense mutation was responsible for the synthetic Dyf phenotype in *nphp-4(tm925);osm-3(yhw66)* animals, we expressed wild type OSM-3 protein tagged with GFP in the double mutants and analyzed them for dye-filling. Compared to non-transgenic siblings, double mutants expressing OSM-3::GFP are able to uptake dye confirming that the *osm-3(yhw66*) mutation is causing the synthetic Dyf phenotype (Fig 3D).

### *osm-3(yhw66)* in combination with *nphp-4(tm925)* causes distal segment structural defects

In contrast to most of the lines derived from the screen, *nphp-4(tm925);osm-3(yhw66)* mutants have correctly fasciculated cilia on the amphid and phasmid neurons (Fig 1C). However, our analysis of cilia morphology using XBX-1::tdTomato reveals its localization is either being restricted to the cilium’s middle segment or cilia were specifically lacking distal segments (Fig 1C and S1 Fig). Distal and middle cilia segments can be identified by the microtubule architecture being singlets or doublets, respectively. Ultra-structural analysis of the cilia in *nphp-4(tm925);osm-3(yhw66)* mutants by transmission electron microscopy (TEM) confirms that the double mutant cilia lack distal segments while maintaining normally formed middle segments (Fig 3E). This is similar to what is seen in *osm-3(mn357)* null mutants [28, 29, 34]. *Osm-3(yhw66)* single mutants were also analyzed; and have properly formed distal segments (Fig 3E).

### *osm-3(yhw66)* has a specific genetic interaction with the mutation in *nphp-4*

**Fig 4:**
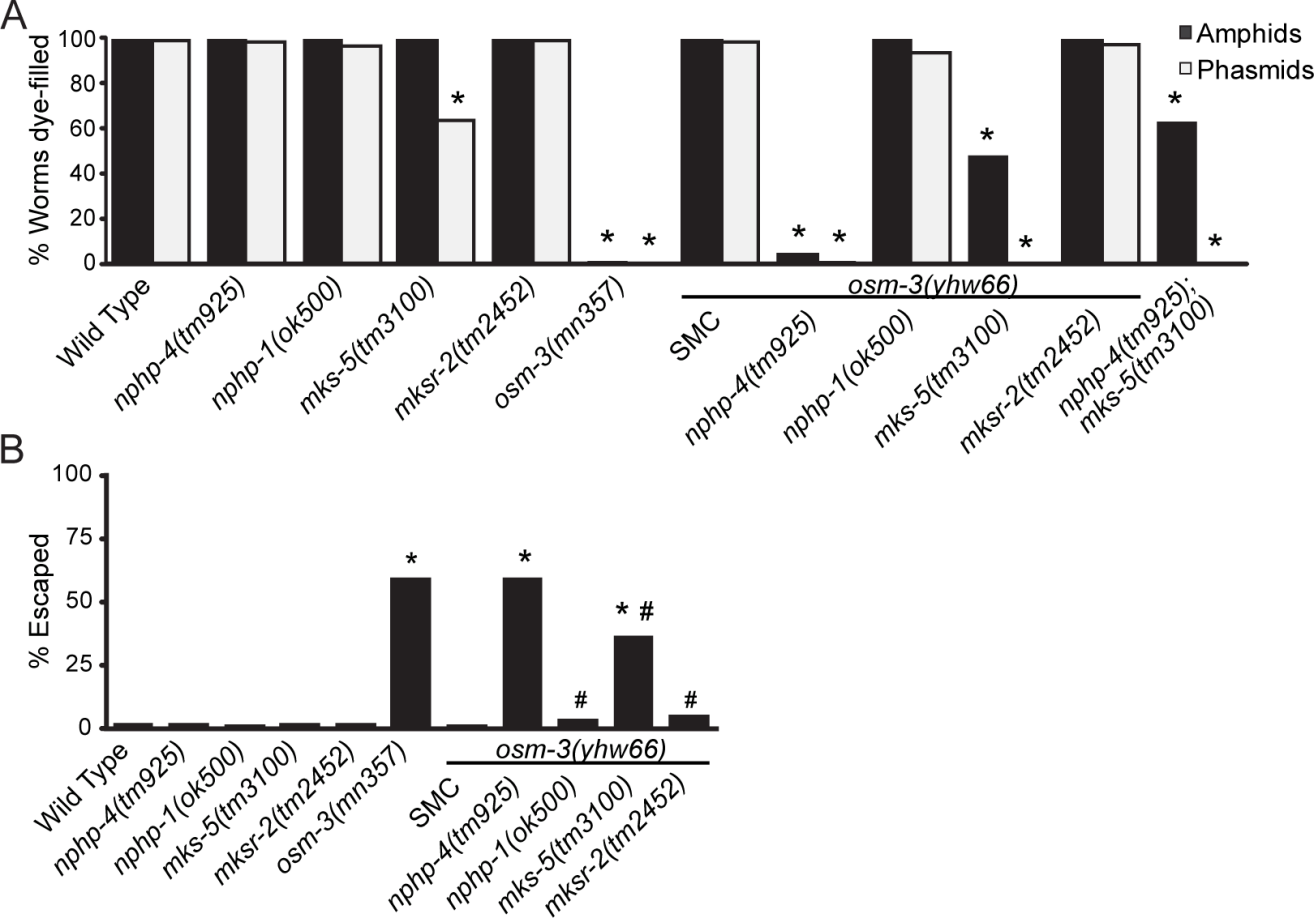
*Osm-3(yhw66)* interaction with the TZ is specific to *nphp-4*. (A) Dye-filling in amphid and phasmid neurons of the indicated TZ mutant and compound mutant strains testing the specificity of the *nphp-4(tm925)* and *osm-3(yhw66)* genetic interaction. Genetic interactions resulting in a Dyf phenotype are evident only in *osm-3(yhw66);nphp-4(tm925)* and *osm-3(yhw66);mks-5(tm3100)* strains. SMC, *osm-3(ywh66)* single mutant control. n = 3 trials with at least 100 animals per trial. * = p<0.05 as compared to SMC. (B) Analysis of the osmotic avoidance behavior of the indicated strains combining the *osm-3(yhw66)* allele with TZ mutations. n = 6 trials with at least 50 animals per trial. * = p<0.05 as compared to SMC. # = p<0.05 as compared to *nphp-4(tm925);osm-3(yhw66)*.

As *osm-3(mn357)* null mutants are Dyf on their own, we also evaluated the synthetic nature of the Dyf phenotype of the *nphp-4(tm925);osm-3(yhw66)* mutant. We crossed *nphp-4(tm925);osm-3(yhw66)* double mutants to wild-type worms to remove the original *nphp-4(tm925)* background mutation. In contrast to the *osm-3(mn357)* null worms, *osm-3(yhw66)* single mutants do not have an obvious Dyf phenotype in either their amphid or phasmid neurons indicating the Dyf phenotype is dependent on the *nphp-4(tm925)* mutant background (Figs 3D and 4A).

To determine the specificity of the genetic interaction between *nphp-4(tm925)* and *osm-3(yhw66)*, the *osm-3(yhw66)* allele was crossed with mutations in several other NPHP or MKS TZ genes and analyzed for the presence of Dyf and Osm phenotypes. No Dyf or Osm phenotypes were present in *osm-3(yhw66);nphp-1(ok500)* or *osm-3(yhw66);mksr-2(tm2452)* mutants; however, there were partial Dyf and Osm phenotypes observed in the *osm-3(yhw66);mks-5(tm3100)* mutants (Fig 4A and 4B). The phenotypes observed in the cross with *mks-5(tm3100)* is expected due to a known role for MKS-5 in localization of NPHP-4 in the TZ (Fig 2I) [25].

### Assessing the consequence of the S316F on OSM-3 function

To begin analyzing the consequence of the mutation on OSM-3 function, we first determined whether there was a difference in anterograde IFT velocity in the middle and distal segments of phasmid cilia using DYF-11::GFP, the homolog of mammalian IFT54 complex B protein. Measuring the anterograde IFT velocity using the IFT-B complex protein (DYF-11::GFP) will account for changes in velocity being due to variations in cargo loading or cargo interactions with the OSM-3 mutations.

**Fig 5:**
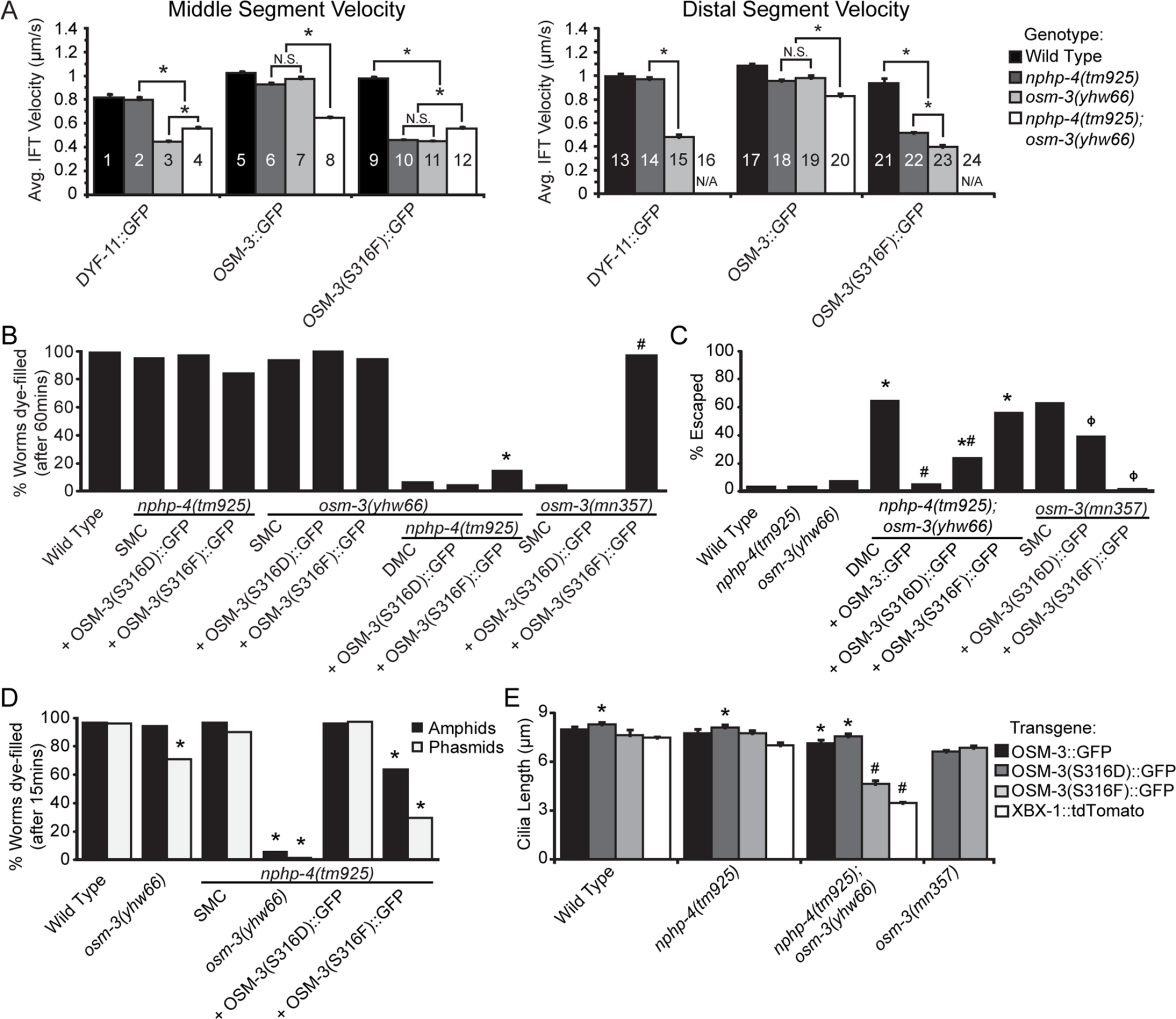
S316 may function as a potential phosphorylation site regulating OSM-3 localization and function. (A) Kymograph data for IFT velocity in middle and distal segments of worms expressing DYF-11::GFP, OSM-3::GFP and OSM-3(S316F)::GFP. N.S. = not significant, * = p<0.05 (B) Analysis of dye-filling in the indicated strains to determine the function of the potential phospho-null OSM-3(S316F)::GFP and phospho-mimetic OSM-3(S316D)::GFP forms of OSM-3. SMC, single mutant control. DMC, double mutant control. N = 3 trials with at least 100 animals per trial. * = p<0.05 as compared to DMC, # = p<0.05 as compared to SMC *osm-5(mn357)*. (C) Analysis of the osmotic (Osm) avoidance behavior in the indicated strains to determine the functional consequence of the phospho-null OSM-3(S316F)::GFP and phospho-mimetic OSM-3(S316D)::GFP mutations. DMC, double mutant control. n = 3 trials with at least 50 animals tested per trial. * = p<0.05 as compared to *nphp4(tm925)*, # = p<0.05 as compared to DMC. (D) Stringent analysis (15 minute) of the Dyf phenotypes in the indicated strains to analyze possible dominant effects of the putative phospho-null OSM-3(S316F)::GFP. * = p<0.05 as compared to *nphp4(tm925)*. (E) Analysis of phasmid cilia length in wild type, *nphp-49tm925)*, and *nphp-4(tm925);osm-3(yhw66)* mutants expressing OSM-3::GFP, OSM-3(S316F)::GFP, or OSM-3(S316D)::GFP transgenes. * = p<0.05 as compared within genotype, # = p<0.05 as compared to *nphp-4(tm925)* expressing same transgene

DYF-11::GFP anterograde IFT velocity in the middle segment of *osm-3(yhw66)* and *nphp-4(tm925)*;*osm-3(yhw66)* mutants was significantly retarded as compared to the wild-type or *nphp-4(tm925)* backgrounds (Fig 5A bars 1–4). These data suggest the retardation of DYF-11::GFP in the *osm-3(yhw66)* and *nphp-4(tm925);osm-3(yhw66)* mutants is caused by the *osm-3(yhw66)* mutation acting independently of the *nphp-4(tm925)* mutation. DYF-11::GFP velocity in the middle segment in the *nphp-4(tm925)*;*osm-3(yhw66)* double mutant background is increased and statistically different from the *osm-3(yhw66)* mutant alone (Fig 5A bar 3 and 4) indicating that loss of *nphp-4* does have an impact on anterograde IFT velocity. The cause of this increase in the anterograde IFT velocity associated with loss of *nphp-4* is not known, but is also observed in the IFT velocity in the middle segment when measured using the OSM-3(S316F)::GFP (Fig 5A bars 11 and 12). A possible explanation based on NPHP-4 functioning as part of a ciliary gate is that in *nphp-4* mutants there is a negative influence on IFT entering the cilium.

Anterograde IFT velocity was next measured in the distal segment where OSM-3 is the sole kinesin responsible for transporting IFT proteins and thus would no longer be influenced by the rate of Kinesin II. A similar retardation of DYF11::GFP IFT velocity was observed in the distal segment of the *osm-3(yhw66)* mutants (Fig 5A, bars 13 and 14 vs 15). Anterograde IFT velocity could not be analyzed in the distal segment of the *nphp-4(tm925);osm-3(yhw66)* double mutants as they fail to form this segment (Fig 5, bar 16).

IFT velocity of wild type OSM-3 tagged with GFP was also analyzed in the same genetic backgrounds. Consistent with the DYF-11::GFP data, OSM-3::GFP IFT velocity was unaffected by the *nphp-4(tm925)* mutation in the middle (Fig 5A bars 5 and 6) and distal segments (Fig 5A bars 17 vs 18). Expression of OSM-3::GFP was able to rescue the IFT velocity in the *osm-3(yhw66)* mutants in the middle segment (Fig 5A, bar 7 vs 5 and 3) and the distal segment (Fig 5A, bars 19 vs 17 and 15). Unexpectedly, OSM-3::GFP did not restore the IFT velocity to wild type levels in the context of a double *nphp-4(tm925);osm-3(yhw66)* mutant in the middle (Fig 5A, bar 8 vs 5–7) or distal segments (Fig 5A, bar 20 vs 17–19). These data indicate that the presence of the endogenous mutant OSM-3(S316F) has a detrimental effect that cannot be fully rescued by overexpressing wild type OSM-3::GFP in the *nphp-4(tm925);osm-3(yhw66)* double mutant background, but can in the *osm-3(yhw66)* background alone (Fig 5A Bars 7 vs 8 and 19 vs 20). The formation of heterodimers between wild type and S316F mutant OSM-3 proteins or OSM-3(S316F) homodimers whose function is perturbed by loss of *nphp-4* could be a possible explanation for this observation. At this point we do not know whether there is a difference in anterograde IFT velocity in mutant homodimers versus wild type-mutant heterodimers. The increase in the wild type OSM-3 dimers that form in the OSM3::GFP overexpressing strains would outnumber the mutant homodimers or mutant-wild type heterodimers reducing the detrimental effect on IFT resulting from the absence of *nphp-4* and lead to this partial rescue of the IFT velocity. A possible explanation for this observation could be that the loss of *nphp-4* could perturb the function of wild type OSM-3 and OSM-3(S316F) mutant heterodimers. Thus diminishing the IFT velocity of a subpopulation of particles being measured and preventing the full restoration of IFT velocities.

Finally, we analyzed the anterograde IFT velocity of the *osm-3(yhw66)* allele by expressing OSM-3(S316F)::GFP. As expected, distal segments are not restored in the *nphp-4(tm925);osm-3(yhw66)* double mutants overexpressing OSM-3(S316F)::GFP, so anterograde IFT velocity could not be analyzed in this region of the cilium (Fig 5A, bar 24). In the middle segment, IFT velocity analyzed using OSM-3(S316F)::GFP expressed in the *osm-3(yhw66)* and *nphp-4(tm925);osm-3(yhw66)* mutants were greatly retarded compared to its expression in wild type controls (Fig 5A bars 11 and 12 vs 9, and 23 vs 21) and were not statistically different from the IFT velocity of DYF-11::GFP in the same backgrounds (Fig 5A, bars 3, 4, and 15). Since the velocity of IFT were unaffected by overexpression of OSM-3(S316F)::GFP in wild type *C*. *elegans* (Fig 5A bars 9 and 21 vs 5 and 17) there is no overt dominant negative effect of the OSM-3(S316F) mutation. In contrast to the effects in the wild type line, overexpression of the OSM-3(S316F)::GFP in the *nphp4(tm925)* mutant background caused a large drop in IFT velocity (Fig 5A bars 10 and 22 vs 9 and 21) similar to that observed in *osm-3(yhw66)* and *nphp-4(tm925);osm-3(yhw66)* double mutants (Fig 5A bars 10 and 22 vs 11,12, and 23). Since this effect of OSM-3(S316F)::GFP on IFT is not observed when expressed in the wild type background, the result is likely caused by the same detrimental effects that losing *nphp-4* has on wild type OSM-3::GFP when heterodimerized with OSM-3(S316F) protein. From this analysis we conclude that there is an epistatic genetic interaction that occurs when there is a mutant and wild type allele of *osm-3* present in the context of the *nphp-4* mutant background. Further, the severity of the effect appears to be proportional to the amount of mutant allele present (e.g. endogenous versus overexpression from the transgene).

### The S316F mutation affects a putative phosphorylation domain important for regulating OSM-3

To assess how the S316F mutation may affect OSM-3 function, the domain in OSM-3 around the mutation was analyzed using a Group-based Prediction System [35]. Based on this computational analysis, we predict that the S316F mutation may be altering a phosphorylation site for Polo-like Kinase 1, Casein Kinase I, and G protein-coupled receptor kinase (GRK)/Beta adrenergic receptor kinase (BARK) group of kinases. To explore this possibility, an OSM-3(S316D)::GFP construct was generated that would substitute a phospho-mimetic amino acid for the original serine residue, S316D. The function of the potential phospho-null (S316F) and phospho-mimetic (S316D) OSM-3 was determined by analyzing their ability to rescue the dye-filling defects (Dyf) in *nphp-4(tm925);osm-3(yhw66)* and *osm-3(mn357)* null backgrounds. Neither S316F nor S316D are able to restore dye-filling in the *nphp-4(tm925);osm-3(yhw66)* background (Fig 5B). However, the phospho-null OSM-3(S316F) is fully capable of rescuing the Dyf phenotype in the *osm-3(mn357)* null mutants, indicating that OSM-3(S316F) is functional as long as there is a wild type *nphp-4* allele.

To further assess functionality of the OSM-3 mutations, their ability to rescue the osmotic avoidance (Osm) defect of the *nphp-4(tm925);osm-3(yhw66)* and *osm-3(mn325)* mutants was evaluated. Wild type OSM-3::GFP fully rescues the Osm defect in *nphp-4(tm925);osm-3(yhw66)* mutants (Fig 5C). As with the Dyf phenotype, OSM-3(S316F)::GFP is not able to rescue the Osm phenotype in *nphp-4(tm925);osm-3(yhw66)* (Fig 5C); however, it rescued the *osm-3(mn357)* null mutants. Thus in the presence of NPHP-4, the OSM-3(S316F) is fully functional despite retarded anterograde IFT velocity. In contrast, OSM-3(S316D)::GFP partially rescued the *nphp-4(tm925);osm-3(yhw66)* and *osm-3(mn325)* null phenotype regardless of the *nphp-4* mutation(Fig 5C).

The differential effect on anterograde IFT velocity caused by expressing OSM-3(S316F)::GFP in wild type and *nphp-4(tm925)* mutants (Fig 5A, bars 10 and 22 vs 9 and 21) suggest that OSM-3(S316F) may have a negative effect on wild type OSM-3 in certain genetic backgrounds. This is also evident in the failure of OSM-3::GFP to fully rescue the IFT rates in *nphp-4(tm925);osm-3(yhw66)* double mutants (Fig 5A bars 6 vs 8) compared to the full rescue in *osm-3(yhw66)* mutants (Fig 5A bars 5 vs 7). To further assess the effect OSM-3(S316F) has over wild type OSM-3 in an *nphp-4* mutant background, we conducted a more stringent 15 minute dye-filling assay (typically done for 40 minutes) in *nphp-4(tm925)* single mutants expressing OSM-3(S316F)::GFP and OSM-3(S316D)::GFP. In the 15 minute assay, the *osm-3(yhw66*) mutant alone does show a mild Dyf phenotype in the phasmid neurons, but not the amphid neurons (Fig 5D). Similar to what is shown in the longer dye-filling assay there is no Dyf phenotype evident in the *nphp-4(tm925)* mutants alone and a strong Dyf phenotype in *nphp-4(tm925);osm-3(yhw66)* double mutants (Fig 5D). However, expression of OSM-3(S316F) in the *nphp-4(tm925)* mutant results in a greatly exacerbated Dyf phenotype present in both phasmid and amphid neurons (Fig 5D). This is not observed in the longer dye-filling assay (Fig 5B) and further supports the idea that OSM-3(S316F) can interfere with endogenous OSM-3 function when NPHP-4 is mutated.

As an additional approach to assess the importance of the S316 residue on OSM-3 function, we evaluated the effect of each mutation on cilia length using XBX-1::tdTomato as a ciliary marker. Phasmid cilia length was compared in wild type, *nphp-4(tm925)*, *nphp-4(tm925);osm-3(yhw66)*, and *osm-3(mn357)* backgrounds expressing the OSM-3 transgenes or XBX-1 alone as a control. The average phasmid cilia length of wild type and *nphp-4* mutants is approximately 7.5μm and was unchanged by expressing wild type OSM-3, OSM-3(S316D), or OSM-3(S316F) (Fig 5E). Thus, while expression of OSM-3(S316F)::GFP in an *nphp-4(tm925)* mutant background does cause a Dyf phenotype (Fig 5D), it does not have a corresponding impact on cilia length.

Cilia length was restored in the *nphp-4(tm925);osm-3(yhw66)* double mutants that expressed wild type OSM-3 as well as OSM-3(S316D). In contrast, expression of the OSM-3(S316F) transgene in *nphp-4(tm925);osm-3(yhw66)* mutants is not able to restore cilia length (Fig 5E). We also analyzed the ability of OSM-3(S316F)::GFP and OSM-3(S316D) to rescue cilia length of *osm-3(mn357)* null mutants. In both cases cilia length was restored to near wild type controls further indicating that the proteins from both alleles are functional with regards to distal cilia assembly. This increase in cilia length of *nphp-4(tm925);osm-3(yhw66)* and *osm-3(mn357)* null mutants expressing OSM-3(S316D) is unexpected in light of OSM-3(S316D) inability to restore dye filling in the double mutants (Fig 5B).

### S316 is an important site regulating OSM-3 localization and function

**Fig 6:**
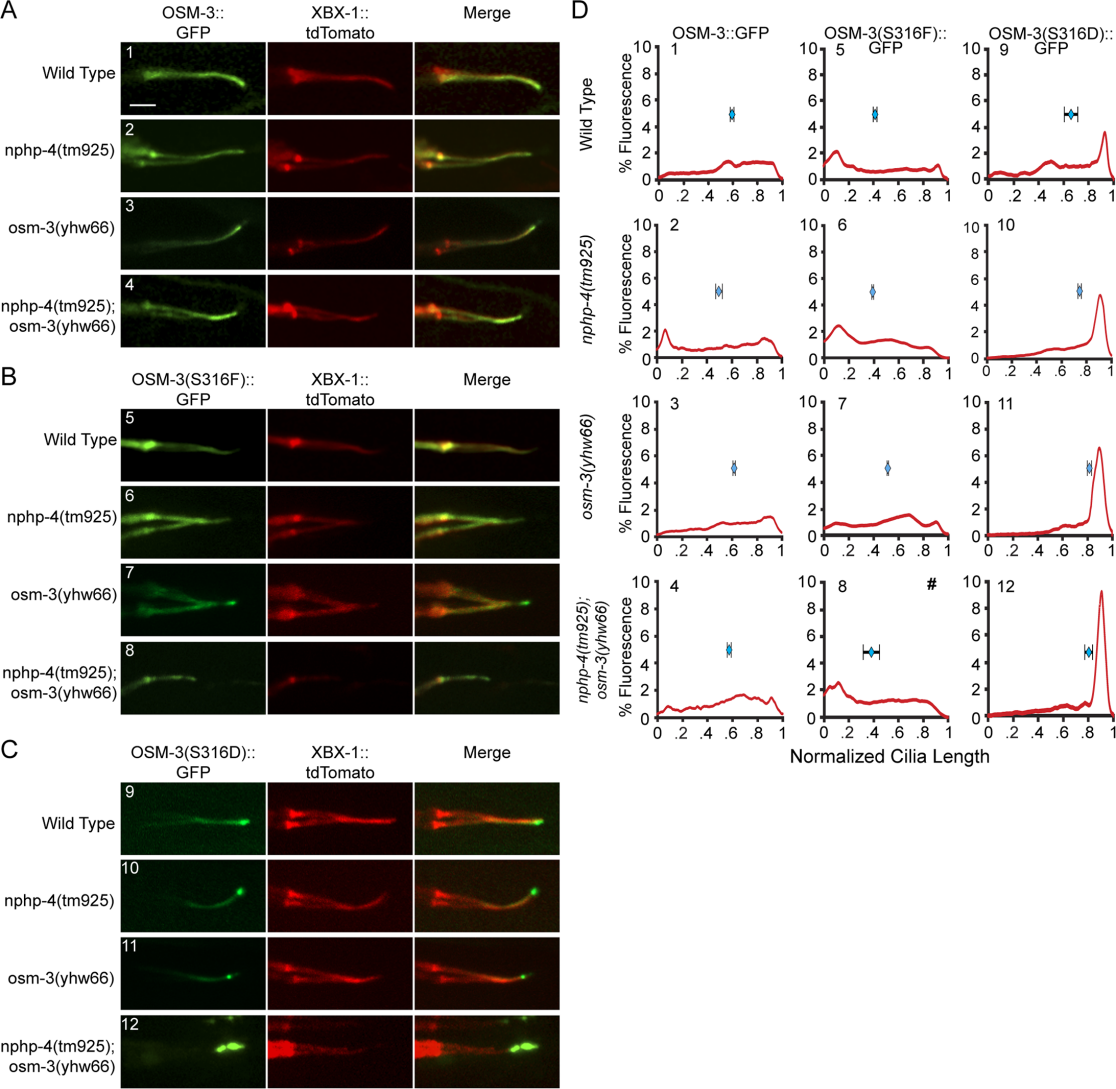
The effects of the phospho-null and phospho-mimetic mutation and genetic background on OSM-3 localization. Representative confocal microscopy images of phasmid cilia of animals co-expressing the XBX-1::tdTomato cilia marker protein and (A) OSM-3::GFP, (B) OSM-3(S316F)::GFP, (C) OSM-3(S316D)::GFP. (D) Line scans of phasmid cilia quantifying percent of the total fluorescence along the cilia axoneme of the indicated OSM-3 transgenes. Cilia length and fluorescence was normalized across genotypes. (#)Note that the *nphp-4(tm925);osm-3(yhw66)* (8) worms lack distal segments and thus have a shorter cilium. The mean distribution of fluorescence intensity along the axoneme is represented by blue diamonds with SEM above each graph. Scale bar 3μm.

To explore the possibility that S316 is an important NPHP-4 dependent regulatory site in OSM-3 we expressed OSM-3::GFP, OSM-3(S316F)::GFP, and OSM-3(S316D)::GFP in each mutant background and quantified fluorescence intensity along the cilium using line scans (note: cilia length was standardized across the genetic lines and that *nphp-4(tm925);osm-3(yhw66)* mutants have shorter cilia that is not reflected in the line scan). In wild type, *nphp-4(tm925)*, and *osm-3(yhw66)* animals OSM-3::GFP localizes along the axoneme with a slight increase in the distal segments (Fig 6A and 6D). OSM-3(S316F)::GFP was similar to OSM-3::GFP in that it localizes throughout the cilium, but in most worms it concentrates more at the cilia base (Fig 6B and 6D). Since OSM-3(S316F)::GFP is able to localize in cilia of the *nphp-4(tm925);osm-3(yhw66)* double mutants the lack of distal cilia segments is not directly due to an inability of OSM-3(S316F)::GFP to enter the cilium.

The localization of OSM-3(S316D)::GFP differs dramatically from that of OSM-3(S316F)::GFP or OSM-3::GFP, and predominantly localizes to the distal tip of the cilium (Fig 6C and 6D). In the *nphp-4(tm925)*:*osm-3(yhw66)* double mutant background the accumulation is further exacerbated. The accumulation at the tip suggests that the putative phospho-mimetic form does not undergo retrograde transport efficiently, which we are not able to detect in kymograph analysis. Strikingly, OSM-3(S316D)::GFP is able to restore distal cilia segment formation in *nphp-4(tm925);osm-3(yhw66)* mutants as visualized with the XBX-1::tdTomato cilia marker (Fig 6C); despite not rescuing the Dyf phenotype (Fig 5B) and only partially correcting the Osm defect (Fig 5C).

**Fig 7:**
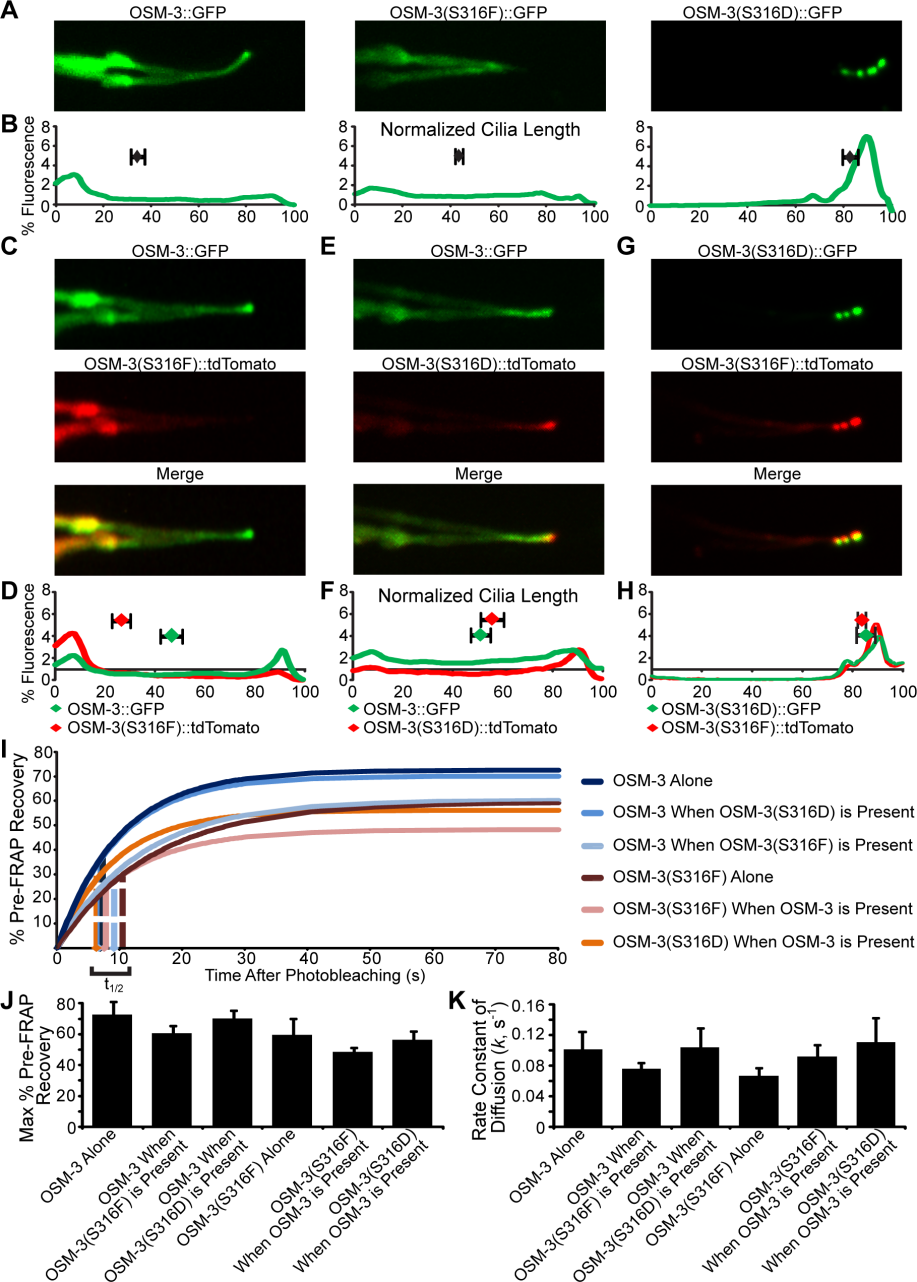
The effects of co-expressing the OSM-3 variants in *osm-3(mn357)* null mutants on localization, ciliary entry and ciliary recovery. (A) Representative confocal images of phasmid cilia of *osm-3(mn357)* mutant animals expressing either OSM-3::GFP, OSM-3(S316F)::GFP, or OSM-3(S316D)::GFP. (B) Line scans of phasmid cilia quantifying percent of the total fluorescence along the cilia axoneme from the indicated OSM-3 transgenes. Cilia length and fluorescence was normalized across genotypes. The mean distribution of fluorescence intensity along the axoneme is represented by blue diamonds with SEM above each graph. Representative images and line scans of *osm-3(mn357)* mutants expressing (C-D) OSM-3::GFP and OSM-3(S316F)::tdTomato, (E-F) OSM-3::GFP and OSM-3(S316D)::tdTomato, and (G-H) OSM-3(S316D)::GFP and OSM-3(S316F)::tdTomato. For all line scans cilia length and fluorescence was normalized across genotypes. The mean center of distribution of fluorescence intensity along the axoneme and SEM is indicated by diamonds and bar above each graph. (I) FRAP results of *osm-3(mn357)* mutant animals expressing or co-expressing the indicated OSM-3 variants. Dotted line = t_1/2_ for fluorescence recovery. (J) Quantification of percent pre-FRAP fluorescence recovery levels of *osm-3(mn357)* mutant animals expressing the indicated OSM-3 variants. (K) Diffusivity value (k (s^-1^)) of fluorescence after FRAP analysis in *osm-3(mn357)* mutants expressing the indicated OSM-3 variants.

To assess whether OSM-3, OSM-3(S316F), and OSM-3(S316D) affect one another transgenic strains of *C*. *elegans* were generated to singly express or co-express OSM-3 variants in an *osm-3(mn357)* null background (Fig 7). OSM-3 and OSM-3(S316F) localize throughout cilia when expressed on their own in the *osm-3(mn357)* background, while OSM-3(S316D) accumulates at the ciliary tip (Fig 7A). When OSM-3::GFP and OSM-3(S316F)::tdTomato are co-expressed, OSM-3::GFP localized throughout the cilia, at the ciliary tip, and at the ciliary base; while OSM-3(316F)::tdTomato accumulated largely at the base (Fig 7C and 7D). This could be the result of OSM-3::GFP homodimers entering the cilia more efficiently than OSM-3(S316F)::tdTomato homodimers. Concurrent expression of OSM-3 and OSM-3(S316D) resulted in relatively even dispersal of OSM-3 throughout the entire cilia with a tendency to reduce levels at the of OSM-3::GFP at the base and more accumulation at the tip along with OSM-3(S316D) (Fig 7D and 7E). This accumulation of OSM-3(S316D) at the tip when co-expressed with wild type OSM-3 was less severe than the accumulation of OSM-3(S316D) without overexpression of OSM-3 (Fig 7A and 7E and 7F). When OSM-3(S316F) and OSM-3(S316D) were co-expressed they both heavily accumulated at the ciliary tip (Fig 7F and 7G). These data suggest that OSM-3(S316D) is able to heterodimerize with OSM-3(S316F) and travel out to the tip as we lose nearly all localization of OSM-3(S316F) at the base. Retrograde transport of OSM-3(S316D)::GFP or OSM-3(S316F)::tdTomato from this tip localization was difficult to detect suggesting they may be anchored at this position. One interpretation of these data is that the inefficient entry observed for the OSM-3(S316F) dimers (Fig 7C) indicate a possible requirement for at least one of the OSM-3 proteins in the dimers to be phosphorylated while de-phosphorylation of both proteins may be required for effective retrograde transport from the tip.

To determine if altering the S316 residue is important for regulating ciliary entry and accumulation, FRAP (fluorescence recovery after photobleaching) experiments were performed on cilia in worms expressing or co-expressing OSM-3, OSM-3(S316F), and OSM-3(S316D)(Fig 7I). OSM-3::GFP in a *osm-3(mn357)* background recovers at k = 0.101s^-1^. In agreement with our prediction on phosphorylation, OSM-3(S316F) recovers fluorescence after photobleaching slower than OSM-3::GFP at k = 0.067s^-1^ (Fig 7I and 7K). We were unable to assess recovery of OSM-3(S316D)::GFP expressed by itself in part because there is no reservoir of OSM-3(S316D)::GFP at the base of the cilium from which the recovery would occur. Rather FRAP analysis in the OSM-3(S316D)::GFP expressing worms would likely measure rates of protein synthesis, transport along the dendrite to the base of the cilium, and docking at the transition zone.

The effect of simultaneous expression of OSM-3(S316F)::tdTomato or OSM-3(S316D)::tdTomato with OSM-3::GFP was also determined using FRAP. The data indicate there is no appreciable difference in diffusivity (how readily the OSM-3 variants enter the cilium) between OSM-3 and OSM-3(S316D) when expressed together (Fig 7I and 7K); however, the expression of OSM-3-(S316F) tended to retard either OSM-3 or OSM-3(S316D) rates of entry in agreement with the putative role for phosphorylation in regulating entry.

The shortfall in percentage of recovery from the 100% pre-FRAP levels of the OSM-3 isoforms is explained by an immobile fraction of protein in the cilia that cannot easily be replenished (Fig 7J). The reduction in total recovery in strains with the OSM-3(S316F) and OSM-3(S316D) compared to OSM-3 alone suggest these variants have amounts of protein that aren't able to readily move back out of the cilium. This correlates with the line-scans and images showing accumulations of protein near the tip of the cilium.

## Discussion

Ciliopathies such as MKS and NPHP have a wide range of phenotypes despite having shared genetic lesions [6, 7, 15, 36]. These data suggest there are likely secondary mutations in the patient’s background that can influence disease presentation. In our previous studies, we did observe such genetic interactions in *C*. *elegans* models that have mutations affecting both *mks* and *nphp* genes. The double *mks;nphp* mutants have severe ciliary defects that are not observed in either of the single mutants alone [12–14]. In the present study, we performed a synthetic Dyf enhancer screen to identify new alleles of genes that functionally interact with an *nphp-4* mutation to modify the severity of the cilia related phenotypes. In this report we identify and confirm four of the ten loci from the screen. Based on previous work, we anticipated that *mks* mutations would be obtained. Thus, the identification of novel alleles in three known *mks* genes (*mks-1*, *mks-2*, and *mks-5*) validates the screening strategy. While the other strains have cilia structural defects that are similar to that of known *mks;nphp* double mutants, the remaining six loci do not correspond to any of the other currently known *mks* genes. Thus, these additional loci once identified will be candidates for genes involved in MKS and/or genetic modifiers of other *ciliopathies*.

Importantly, the analysis of these new *mks* alleles obtained in the screen has further refined the hierarchy of interactions within the MKS module. The new *mks-1* mutation is a more severe allele than the previous *mks-1* mutation. Thus, we were able to demonstrate a role for MKS-1 in the assembly of the MKS module that was not evident in previous studies with the less severe allele [12, 14, 15]. Additionally, the *mks-5(yhw91)* allele obtained in the screen only deletes amino acids from the very C-terminal end of the protein which suggests there are important functional domains within this region, although we do not know at this point whether overall stability of the protein is affected.

Since only three alleles of known *mks* genes (*mks-1*, *mks-2* and *mks-5)* were obtained, these data also indicate the screen is not saturated. This could be because many of the known MKS genes are relatively small and are less likely to be mutagenized efficiently by EMS; or because EMS produces mostly point mutations and such mutations may not be functionally perturbing enough in *mks* genes to produce the synthetic Dyf phenotype with *nphp-4*(*tm925*). In support of this possibility, the point mutations that were identified in *mks* genes (*yhw146*, *yhw128*, and *yhw91*) in the screen occurred at splice donor sites or at the translational start codon. An alternative screen using mutagens such as Trimethyl Psoralen and ultraviolet light to produce more frequent and/or larger deletions may be more effective for saturation. However, an advantage of an EMS screen was the opportunity to uncover novel hypomorphic alleles, which would reveal functionally important domains within these proteins (e.g. OSM-3(S316F)).

While it was anticipated that TZ proteins would be obtained from the screen, the identification of a missense allele in OSM-3 was unexpected. OSM-3 is a kinesin required for the assembly of and for IFT movement along the distal cilia segment [29–31, 37]. The *nphp-4(tm925);osm-3(yhw66)* mutant was unique among those obtained from the screen in that they were the only ones showing normal fasciculation of cilia along with a clear loss of specifically the distal cilia segments. This novel *osm*-3*(yhw66)* allele, while it alters IFT rates, causes minimal structural or functional defects on its own. However, in combination with *nphp-4(tm925)*, it produces cilia structural (loss of distal segments) and functional defects comparable to those seen in *osm-3(mn357)* null mutants [31]. This genetic interaction appears to be specific for *nphp-4* as the *osm-3(yhw66)* allele did not produce this phenotype when in combination with a mutation in another core MKS module protein or another NPHP module protein. It did show partial penetrance of the cilia phenotypes in combination with a mutation in *mks-5*. This result was anticipated based on data showing that loss of MKS-5, in addition to causing mislocalization of all known MKS module proteins, also alters NPHP-4 localization within the TZ [25]. These data suggest a specific genetic link between *nphp-4* and *osm-3* and an indirect effect of MKS-5 on OSM-3.

Previous data have suggested there may be an interaction between *osm-3* and *nphp-4* as OSM-3 was found to accumulate in the dendrite and at the ciliary base in *nphp-4(tm925)* mutants [31, 38]. We also observe OSM-3::GFP accumulation in the *nphp-4(tm925)* mutants and have expanded on the evidence for the interaction between *nphp-4* and *osm-3* with the new *osm-3(yhw66)* allele from the screen.

The data indicate that S316 in OSM-3 is an important and conserved site. Based on computational predictions, S316 is may be a substrate for Polo-like Kinase I, Casein Kinase I (CKI) or beta-adrenergic receptor kinase (BARK) phosphorylation. Previous protein crystallography data of closely related kinesin-1 suggests the S316F mutation is located near the ATP binding pocket of the motor [39]. Thus this mutation could also alter the activation and efficiency of the protein if it impairs ATP binding.

A significant outcome from this study is that a core TZ protein, such as NPHP-4, can have roles not just in regulating entry or retention of proteins in the cilia but it also has an important role in regulating OSM-3 activity and localization within the cilium. How NPHP-4 is regulating OSM-3 function within the cilium is currently unknown. One possibility is that NPHP-4 regulates entry or retention of a kinase or phosphatase in cilia that functions to regulate OSM-3 phosphorylation state. In fact, mutations in several TZ genes in *C*. *elegans* and mammalian cells are known to alter what proteins are present in the cilium [14, 40]. As our data indicate, changes in the putative phosphorylation state of OSM-3 has a significant impact on its localization and on its functional properties. Phosphorylation of OSM-3 at S316 in the middle segment of the cilium may influence its rate of movement toward the tip or its ability to associate with singlet microtubules in the distal segment, while de-phosphorylation at the tip may be needed for its assembly with the retrograde transport machinery for its return back to the base of cilia. We are currently exploring these mechanisms and testing candidate kinases and phosphatase that may be involved.

Although we have not yet demonstrated that S316 is a phosphorylation site, changing the amino acids to mimic a phosphorylated condition has a major impact on OSM-3 function and localization. Phosphorylation events are known to be important in regulating ciliary kinesin activity and for retention of proteins such as polycystin-2 in the cilium [31, 41, 42]. Recent studies indicate that Ca^2+^/calmodulin-dependent protein kinase II (CaMKII) phosphorylates a site near the C-terminus of Kif17, the mammalian homolog of OSM-3, to regulate the interaction between Kif17 and its cargo [43]. In addition, a similar calcium dependent kinase (CrCDPK1) in *Chlamydomonas* phosphorylates Fla8 to regulate its association with the IFT particle (complex B) and kinesin turnaround in cilia [41]. Fla8 is the homolog of mammalian Kif3b and *C*. *elegans* KLP-11. In *C*. *elegans* KLP-11 mediates IFT in the proximal cilia region [44]. In contrast to our data with OSM-3, phosphorylated Fla8 (Fla8-S663D) inhibits entry of kinesin-II in the cilia, whereas unphosphorylated Fla8 (FLA8-S663A) stimulates entry [28, 41]. Kinesin 2a (Kif2a) has recently been shown to localize to the basal body at the base of the cilium when it is not involved in the cell cycle, and when phosphorylated by another ciliary kinase, Polo-like Kinase 1, becomes activated–showing a similar behavior as we observe for OSM-3. Interestingly, this phosphorylation event occurs in a similar conserved sequence of amino acids just after the motor domain as the S316F mutation in OSM-3 [45].

While *osm-3(yhw66)* worms have no overt ciliary defects and OSM-3(S316F) is functional as it rescues *osm-3(mn357)* null mutant cilia phenotypes, OSM-3(S316F) does affect anterograde IFT velocities. This occurs even in the presence of wild type NPHP-4 (Fig 5A, bar 3). While our analyses indicate that OSM-3(S316F) effects on IFT velocities are largely independent of *nphp-4*, in specific context the *nphp-4* mutation does exacerbate these effects. For example, expression of OSM-3::GFP in either *nphp-4(tm925)* or *osm-3(yhw66)* mutants show control IFT velocities (Fig 5A, bars 6 and 7); however, this fails to occur when OSM-3::GFP is expressed in the *nphp-4(tmn925);osm-3(yhw66)* double mutants (Fig 5A, bar 8).

A possible explanation for many of the effects we observe on IFT would be that in NPHP-4 wild type backgrounds, OSM-3(S316F) homodimers are not transported as efficiently across the TZ as wild type OSM-3, thus leading to a preference of wild type OSM-3 homodimers or possibly OSM-3(S316F)-OSM-3 wild type heterodimers in the cilia. When NPHP-4 is absent, OSM-3(S316F) is no longer impaired from crossing the TZ. Thus, the IFT velocity would be dictated by the dose of each allele and whether NPHP-4 is present. In the absence of NPHP-4, the mutant homodimers with their slower movement would significantly impair IFT speeds. This would also help explain why overexpression of OSM-3(S316F)::GFP in the *nphp-4(tm925)* background has such a profound effect on IFT (Fig 5A bar 10) and why expression of OSM-3::GFP in *nphp-4(tm925);osm-3(yhw66)* double mutants is not able to restore rates to expected *nphp-4(tm925)* level (Fig 5A bar 8).

The OSM-3(S316F) mutation also affects localization of the protein. While OSM-3(S316F) does enter the cilium, it tends to accumulate more in the basal region of cilia than OSM-3::GFP and this effect is increased in *nphp-4* mutants. In contrast to what is observed for OSM-3(S316F), when the putative phospho-mimetic OSM-3(S316D) is expressed, it accumulates in the distal tip of the cilium. The distal tip accumulation is increased in lines lacking *nphp-4* and is further augmented in the *nphp-4;osm-3(yhw66);* double mutants. Thus, the presence of a wild type OSM-3 is able to partially reduce the distal tip accumulation. Further simultaneous expression of OSM-3::GFP and OSM-3(S316D)::tdTomato show OSM-3::GFP accumulates at the ciliary tip more than when expressed by itself and that the tip accumulation of OSM-3(S316D)::tdTomato is reduced compared to when OSM-3(S316D) is expressed alone. Many of these effects may be explained through forming a heteroduplex between wild type OSM-3 and OSM-3(S316D), that is increasing OSM-3 ciliary tip localization and increasing OSM-3(S316D) retrograde transport, although we were unable to reliably detect OSM-3(S316D) retrograde movement from the cilia tip in these lines.

Interestingly, OSM-3(S316D) is able to restore the distal segments in the *nphp-4;osm-3(yhw66)* double and *osm-3(mn325)* null mutants, while OMS-3(S316F) requires wild type NPHP-4. But despite the ability to restore cilia length, OSM-3(S316D) did not rescue the Dyf phenotype and only partially corrected the Osm phenotype. This is unexpected as in most cases restoring cilia length also corrects the Dyf phenotype. An exception to this are Bardet-Biedl Syndrome (*bbs*) mutations that have a Dyf phenotype but have no loss in cilia length [46].

Through FRAP experiments it was determined that OSM-3(S316F) has a slower recovery rate than either OSM-3 or OSM-3(S316D). One possible interpretation of these data is that OSM-3/OSM-3(S316F) heterodimers enter slower than OSM-3 homodimers, but faster than OSM-3(S316F) homodimers. The lower recovery rate of OSM-3(S316F) can also be explained by preferential entry of OSM-3 over OSM-3(S316F). Determining the recovery rate for OSM-3(S316D) when expressed with OSM-3(S316F) or alone was problematic, in part because there is no reservoir of OSM-3(S316D) at the base. These data raise the possible importance of phosphorylation in regulating OSM-3 entry. If the model is correct, OSM-3(S316F) entry would be retarded and it would accumulate at the base while OSM-3(S316D) would enter immediately and would not be retained at the base. While this is observed, a concern with this model is that there is no overt difference in entry rates between OSM-3(S316D) and OSM-3 in the FRAP analysis. We would anticipate that OSM-3 entry would be slower than OSM-3(S316D) since OSM-3 would require a phosphorylation event to mediate its ciliary entry.

Further, OSM-3(S316D) localization at the tip suggests de-phosphorylation may promote retrograde transport back toward the base. This is supported by the data showing that OSM-3(S316D) tip accumulation is reduced when wild type OSM-3 is also expressed. This raised the possibility that de-phosphorylation of one member of the OSM-3/OSM-3(S316D) dimer may promote retrograde movement. Thus, it was unexpected when the level of OSM-3(S316D) at the tip was not reduced by co-expression of OSM-3(S316F) that could not be phosphorylated. Additional studies are underway to explore how phosphorylation and dephosphorylation may regulate entry and return of the OSM-3 complex.

The FRAP data also provides insights into the amount of OSM-3 that is maintained in a mobile or immobile fraction within the cilium. OSM-3(S316F) had the lowest total percentage recovery and also was able to reduce the recovery level of OSM-3, indicating that OSM-3(S316F) has a higher amount in the immobile or non-replaceable fraction. This was most evident in lines co-expressing OSM-3(S316F) and OSM-3(S316D) that had almost exclusive localization at the tip. Recovery in this strain was greatly impaired relative to all the other strains and could not be accurately quantified.

The mammalian homolog of OSM-3 (KIF17) is important for development of the photoreceptor outer segments, a highly modified form of primary cilia that also possess singlet microtubules similar to *C*. *elegans* cilia [47–49] [50]. Here we identified specific mutations that impact OSM-3 activity and localization, many of which were dependent on loss of *nphp-4*. As the potential phosphorylation site S316 in OSM-3 and surrounding sequence are highly conserved in Kif17, it will be important to explore whether a similar genetic interaction occurs in a mammalian context and these studies are currently underway. These data do suggest that background mutations affecting KIF17 could help explain some of the phenotypic variability and the retinal degeneration that occurs in NPHP-4 patients [10, 11, 51]. Based on our data, we suggest that KIF17 should be considered as a strong candidate for a modifier in NPHP patient that present with extra-renal manifestations such as blindness and anosmia.

## Materials and Methods

### General molecular biology methods

General molecular biology was conducted following standard procedures as described [52]. *C*. *elegans* genomic DNA and cloned worm DNA were utilized for PCR amplifications and direct sequencing [52]. PCR conditions and reagents are available upon request. Genome sequencing was performed by the UAB Genomics Core Facility of the Heflin Center for Human Genetics.

### DNA and protein sequence analyses

Genome sequence information was obtained from the National Center for Biotechnology Information (http://www.ncbi.nlm.nih.gov/) or from the Celera Database (http://www.celera.com). Gene sequences were identified using the *C*. *elegans* database Wormbase and references therein (http://www.wormbase.org). Sequence alignments were performed using ClustalW (http://www.ebi.ac.uk/clustalw/).

### Strains

Strains were grown on regular nematode growth media (NGM) using standard *C*. *elegans* growth methods [53] at 20°C unless otherwise stated. The wild-type strain was N2 Bristol. The full list of strains used in the study is available in Table 1. All mutant strains were outcrossed at least three times and genotyped by PCR. The rescue lines were generated using UNC-122::GFP [54] or *rol-6* [55] as a marker to identify transgenic lines.

### Generation of constructs and strains

pcGV6 and pcGV7 Gateway vectors, p328 (XBX-1::TdTomato), and p380 (MKS-5::TdTomato) were described previously [20, 25]. pMH6 (OSM-3::GFP) was generously provided by J. Scholey, University of California, Davis. p389A (OSM-3(S316F)::GFP) was generated by amplifying the 2 kb promoter and entire coding region of *osm-3* from *osm-3(yhw66);nphp-4(tm925)* DNA and inserting the fragment in cGV7 using Gateway cloning technology. All PCR was performed using AccuTaq-LA DNA Polymerase (Sigma, St. Louis, MO) according to manufacturer’s instructions. The resulting constructs were sequenced and injected at 5 ng/μl. For each series of outcrosses the resulting F2 offspring obtained from self-fertilization were screened by PCR to identify strains containing required mutations.

### EMS mutagenesis

Mutagenesis was performed as described previously [56] with modifications. Briefly, a synchronized P_0_ population of young adult *nphp-4(tm925)* mutant worms were grown at room temperature, washed with M9 solution (0.3% KH_2_PO_4_, 0.6% Na_2_HPO_4_, 0.5% NaCl, 1mM MgSO_4_) three times, and placed into a 15-ml conical tube. Worms were concentrated by centrifugation at 2,000 X g and suspended in 2 ml of M9. 2 ml of 60mM EMS (dissolved in M9) solution was added to the 2 ml of worm suspension to reach a final concentration of 30 mM. The vial was rotated for 4 hours at room temperature. Worms were then collected by centrifugation at 2,000 X g and washed three times with M9, rotating between washes. Worms were suspended in 0.5 ml M9 and plated onto NGM plates seeded with OP50 and allowed to recover overnight at 20°C.

The screen for dye-filling defective animals was performed as follows. The day following the mutagenesis, four mutagenized worms (P_0_) were placed on the edge of an *E*. *coli*-seeded 10-cm NGM agar plate, and 8 such plates were made. P_0_ were allowed to lay F1 generation overnight and the next day were transferred onto new plates. This was repeated two more times with the purpose of F1 generation separation by age. Once F1 worms reached adulthood, 50–60 animals from each plate were transferred onto new plates to produce F2 generation. The F1 worms were transferred two more times with the same goal to produce F2 separated by age. As soon as F2 worms on the earliest set of plates reached adulthood, they were collected by centrifugation and dye-filled. Dye-filling defective animals were isolated by visual inspection and manual picking using a Lieca MZ16FA fluorescent microscope. About 4,000 mutagenized haploid genomes and about 500,000 individual F2 worms were screened.

### SNP mapping

To identify the chromosomal location of the genetic lesions responsible for the Dyf phenotypes, a single nucleotide polymorphism (SNP)-based mapping strategy was applied essentially as described [22]. Briefly, 5 hermaphrodites from the established mutant lines were crossed with 20 wild type Hawaiian males. The F1 generation from the cross was dye-filled and 60 normally dye-filled L4 worms were picked from the plates. These L4 worms were plated onto new plates (15 worms per plate) and allowed to self-fertilize. The F2 generation was dye-filled at young adult stage and approximately 50 to 100 Dyf and wild type dye-filled worms were picked for bulk segregate analysis. For interval mapping on LG IV additional SNPs and primer sequences were obtained from Wormbase (http://www.wormbase.org).

### Non-complementation analysis

To determine which of the mutant lines were allelic, we intercrossed males and hermaphrodites from different lines that mapped to the same linkage group. The F1 progeny resulting from each cross was tested for their dye-filling phenotypes in male worms to ensure progeny were from a genetic cross. The presence of nonDyf male progeny indicated complementation. If F1 male progeny displayed only Dyf phenotypes, the mutant lines were considered to be allelic. In cases when double mutant males had mating defects, an alternative strategy was taken; double mutant lines were crossed to *nphp-4(tm925)* males, and F1 heterozygous males were then crossed to other mutant lines which had mutations on the same chromosome. The F1 progeny from these crosses was assessed for dye-filling defective phenotypes. The lack of dye-filling defective male progeny indicated complementation. If any Dyf males were observed, the two mutant lines were considered allelic. Similar mating schematics were applied to determine whether newly isolated alleles were in previously identified *mks* genes. For X-linked mutations, F1 hermaphrodite progeny were analyzed.

### Imaging

Worms were anesthetized using 10 mM Levamisole, immobilized on a 10% agar pad coated with polystyrene beads. Fluorescence imaging was performed using a Nikon 2000U inverted microscope (Melville, KY) outfitted with a PerkinElmer UltraVIEW ERS 6FE-US spinning disk laser apparatus (Shelton, CT). Confocal images were captured every 0.2μm in the z-axis with a 100x (NA 1.39) objective and analyzed with Volocity 6.3 software (Improvision Inc., Waltham, MA). Images were processed into Figs using Photoshop 7.0 and Illustrator CS5 (Adobe Systems, Inc., San Jose, CA). Time laps movies of IFT particles moving along the axoneme were acquired for kymographs at a rate between 3-4fps at a single z-plane for 300 frames.

### Electron microscopy

Electron microscopy on *C*. *elegans* was performed following protocol previously described [57, 58]. Briefly, samples were fixed in 3.5% glutaldehyde and 1% paraformaldehyde in Na-Cacodylate buffer (pH 7.3) for 2.5 hours. Post fixation was performed in 1.5% OsO_4_ for one hour. *En bloc* staining with 1% uranyl acetate was done for 45 minutes. Samples were dehydrated in increasing levels of EtOH and propyline oxide, infiltrated with Embed812 resin, and then embedded in molds with resin. Molds were incubated at 60°C for 2 days. The samples were serial sectioned with a Leica Ultracut UC6 in 80nm sections. Sections were post-stained in 2% aqueous uranyl acetate and Reynold’s lead citrate for five minutes each. Sections were imaged at 80Kv using a FEI Tecnai T12 electron microscope.

### Assays

Dye-filling assays were performed using DiI (Molecular Probes, Carlsbad, CA) as described previously with modifications [33]. In brief, fluorescent dye uptake was performed as described previously [13, 59]. L4 larvae were incubated in Vybrant DiI (Invitrogen; 1:1,000-fold dilution of 1 mM stock in M9 buffer) for 15 or 40 min, allowed to roam on a plate with bacteria for 1 h to clear intestinal dye, and observed by fluorescence microscopy. One hundred worms were picked at random from each strain and analyzed for dye-filling defects in amphid and phasmid neurons using a Lieca MZ16FA fluorescence stereomicroscope. Worms were scored as Dyf if there was no dye detected. In the case of transgenic lines, GFP or Rol marker expressing worms were picked for the assays.

Procedures for osmotic avoidance assay were performed as described in [19] with slight modifications. NGM agar plates of uniform weight were prepared the day before experiments for optimal dryness conditions. An osmotic ring 15 mm in diameter was created on the center of the plates by adding 25–30 μl 8M glycerol spread on the cap of a test tube and then lightly pressed onto the surface of the agar. Glycerol was allowed to soak into agar for 2 to 5 minutes. Worms were rinsed off culture plates into a 15 ml tubes and washed three times with M9 allowed to settle. Then worms were suspended in 50 μl of M9 and transferred to the center of the osmotic ring. Excess liquid was removed by blotting with Kimwipes; once liquid was removed and worms began to move, timing of the trial was started. The total number and number of animals that escaped the osmotic ring after 15 minutes was recorded. Worms that died in the osmotic barrier were counted as escaped. The data are reported as the fraction of worms that escaped the barrier.

### Whole genome sequencing and sequencing data analysis

DNA libraries were prepared according to Illumina's 'Preparing Samples for Sequencing Genomic DNA' protocol (Part # 1003806 Rev. B March 2008). Samples were sequenced using Illumina's GAIIx machine in the laboratory of Dr. Oliver Hobert (Columbia University) or the Heflin Center for Genomic Sciences (University of Alabama at Birmingham). The data were analyzed using MAQGene programs or the Integrative Genomics Viewer [60, 61].

### Statistical analysis

For osmotic avoidance assays comparisons were performed using the Student’s t-test. If the data was not normally distributed, the Mann-Whitney Ranked Sum was used instead. These tests were performed using SigmaStat3.1 software package (Systat Software IC).

Dye-filling experiments were analyzed using a 2x2 chi-square test in R (R foundation for statistical computing, 2014). An FDR postHoc test was completed to account for multiple comparisons. For all tests, p values less than 0.05 were considered significant.

Kymograph experiments were analyzed using a two-way ANOVA with a Tukey’s Post-Hoc test. These tests were performed using R version 3.1.2 software package (R foundation for statistical computing, 2014). For all tests, p values less than 0.05 were considered significant.

Cilia Length experiments were analyzed using a one-way ANOVA with a Tukey’s Post-Hoc test. These tests were performed using R version 3.1.2 software package (R foundation for statistical computing, 2014). For all tests, p values less than 0.05 were considered significant.

### Line scans

Tails of worms were imaged following previously stated protocol. Images were then analyzed using Volocity 6.3 software (Improvision Inc., Waltham, MA). Using Volocity a line was drawn along the phasmid cilia starting 1μm before the base of the cilia into the IFT compartment and ending 0.5μm past the tip of the cilia. The line was drawn based of XBX-1::tdTomato fluorescence. Cilia lengths and fluorescence intensity after background fluorescence was subtracted were normalized. Percent total fluorescence of GFP compared to position along the normalized cilia was calculated using excel.

### FRAP experiments

Worms expressing various forms of OSM-3 were imaged using the protocol above with time-lapse imaging. The phasmid cilia were then bleached (determined by the region at the end of the pre-ciliary compartment to the ciliary tip) leaving the pre-ciliary compartment unbleached. The light path was split using a Dual View (Photometrics) beam splitter to bleach and image both GFP and tdTomato simultaneously. Recovery of fluorescence over 3 minutes was captured at 1–3 frames per second for the first 30 seconds and 0.1 fps afterwards. Background fluorescence was subtracted before analysis. Recovery curves were fit to the one-dimensional diffusion equation F(t) = A*e^(-kt)^, where A is the maximum recovery of florescence in the cilium as compared to pre-bleach levels and *k* is the rate constant of entry into the cilium.

### Cilia length

Tails of worms were imaged following previously stated protocol. Images were then analyzed using Volocity 6.3 software (Improvision Inc., Waltham, MA). A line drawn from the base of the phasmid cilia to the tip was used to measure the length.

## Supporting Information

S1 Fig Structural abnormalities of cilia on phasmid neurons of *nphp-4(tm925)*-dependent synthetic Dyf mutants. Representative confocal images of phasmid (tail) neurons using the XBX-1::TdTomato cilia marker protein in wild-type, *nphp-4(tm925)*, and *nphp-4(tm925)*-dependent synthetic Dyf double mutant lines. Scale, 9μm.
(TIF)

S2 Fig *yhw146* and *yhw91* cause premature termination in MKS-1 and MKS-5, respectively. The *mks-1(yhw146)* G/A substitution (denoted by asterisk) is predicted to disrupt splicing between exons 2 and 3 of the *mks-1* transcript and cause read-through into intron 2 where a stop codon is immediately located. (B) Sanger sequencing of *mks-5(yhw91)* cDNA shows that the nt8247 G/A substitution (denoted by asterisk) affects an exon 15 splice donor site. The use of a cryptic splice donor results in an aberrant transcript with a premature translational termination sequence. (C) The altered *yhw91* transcript is predicted to introduce 17 unique amino acids onto the C-terminus of the truncated MKS-5 protein.
(TIF)

S3 Fig Representative kymographs used to calculate anterograde IFT velocities. Kymograph images of DYF-11::GFP, OSM-3::GFP, and OSM-3(S316F)::GFP in WT, *nphp-4(tm925)*, *osm-3(yhw66)*, and *nphp-4(tm925);osm-3(yhw66*). Numerical data for these experiments can be found in Fig 5. MS = Middle segment, DS = Distal segment.
(TIF)
