# Supplementary figures and images for: A Screen for Modifiers of Cilia Phenotypes Reveals Novel MKS Alleles and Uncovers a Specific Genetic Interaction between osm-3 and nphp-4
Source: PLoS Genet. 2016 Feb 10;12(2):e1005841. doi: 10.1371/journal.pgen.1005841 (PMC4749664; doi:10.1371/journal.pgen.1005841)

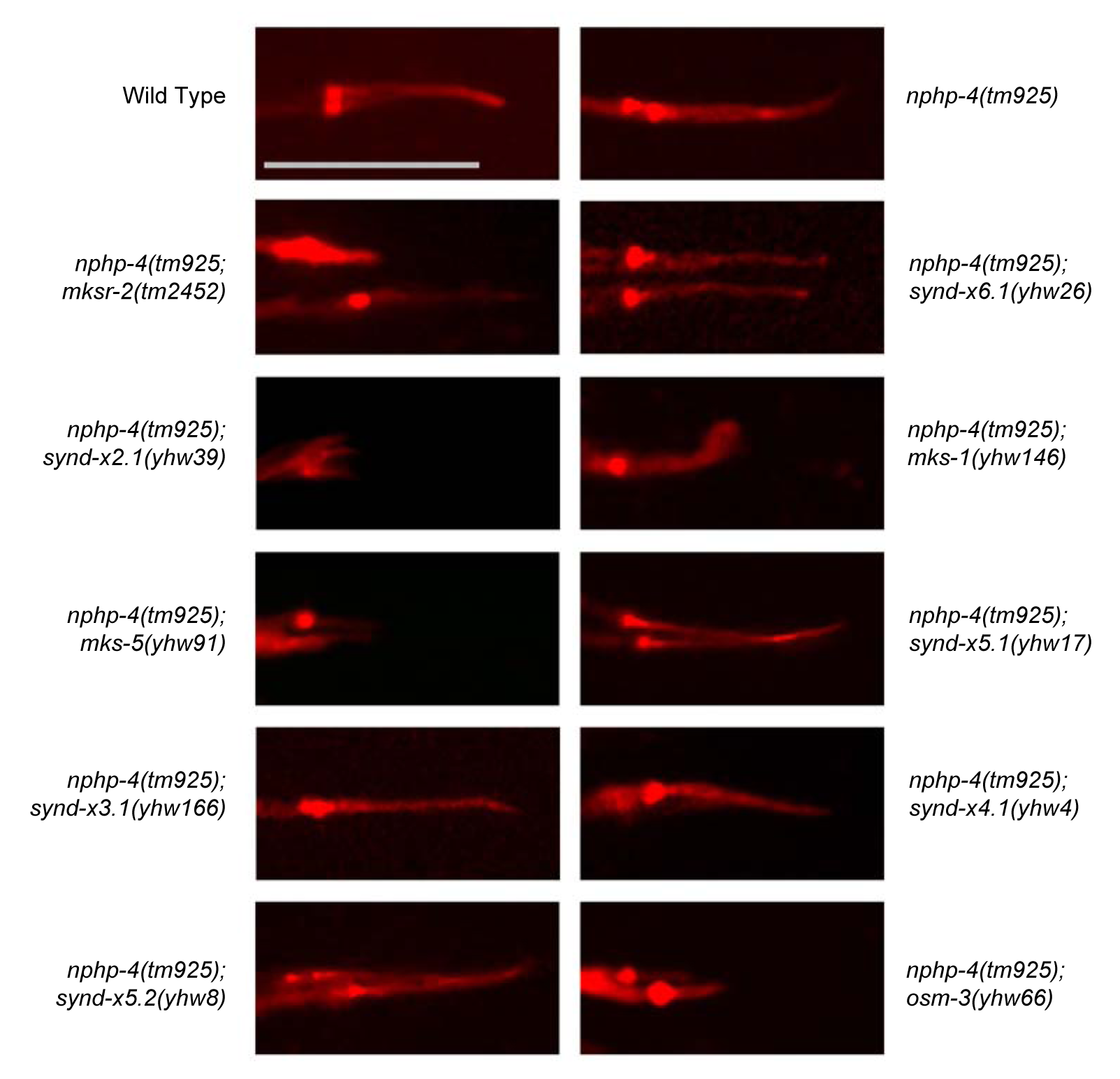

Supplement: S1 Fig — Representative confocal images of phasmid (tail) neurons using the XBX-1::TdTomato cilia marker protein in wild-type, nphp-4(tm925), and nphp-4(tm925)-dependent synthetic Dyf double mutant lines. Scale, 9μm. (TIF) [file pgen.1005841.s001.tif]

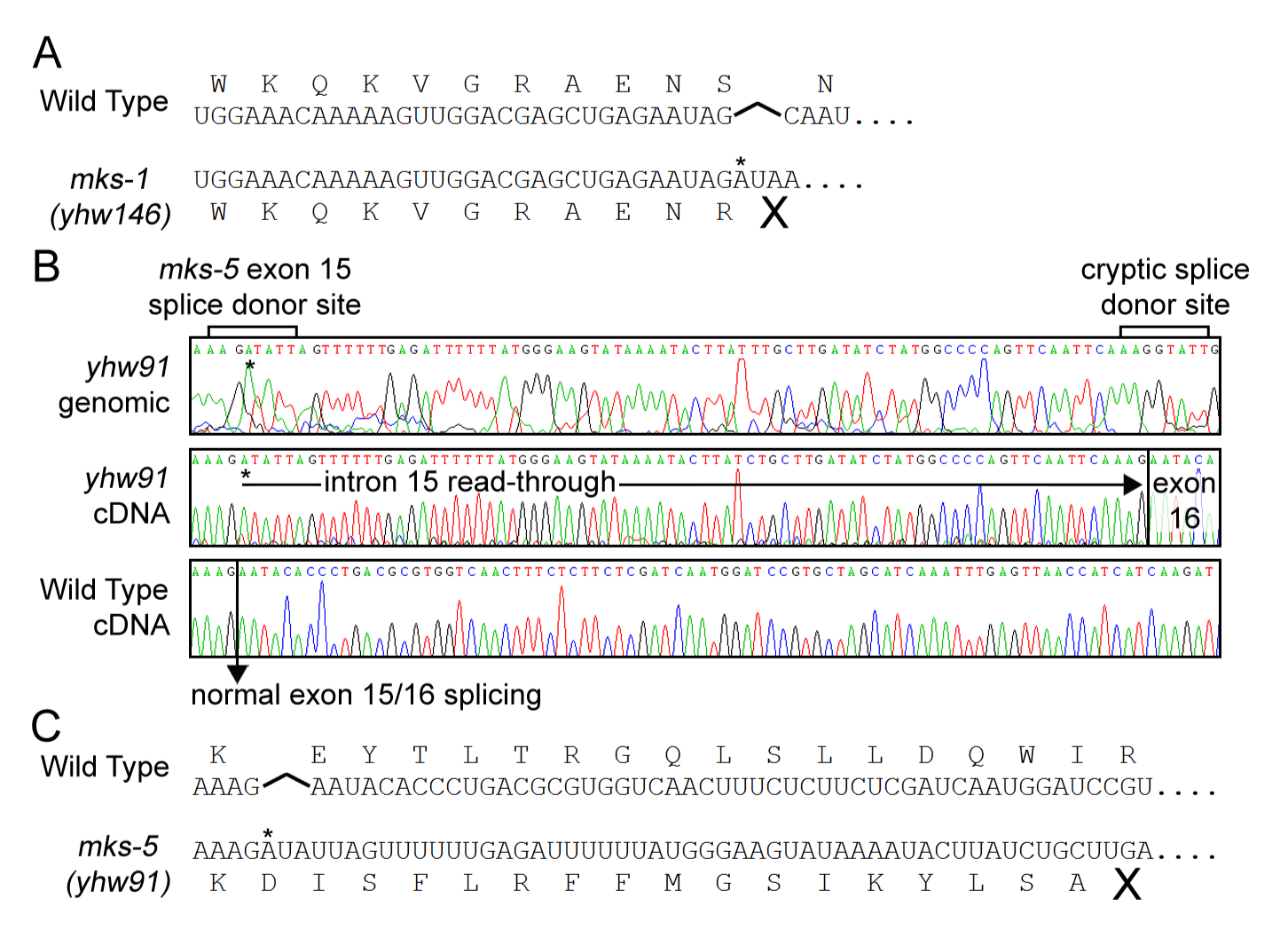

Supplement: S2 Fig — The mks-1(yhw146) G/A substitution (denoted by asterisk) is predicted to disrupt splicing between exons 2 and 3 of the mks-1 transcript and cause read-through into intron 2 where a stop codon is immediately located. (B) Sanger sequencing of mks-5(yhw91) cDNA shows that the nt8247 G/A substitution (denoted by asterisk) affects an exon 15 splice donor site. The use of a cryptic splice donor results in an aberrant transcript with a premature translational termination sequence. (C) The altered yhw91 transcript is predicted to introduce 17 unique amino acids onto the C-terminus of the truncated MKS-5 protein. (TIF) [file pgen.1005841.s002.tif]

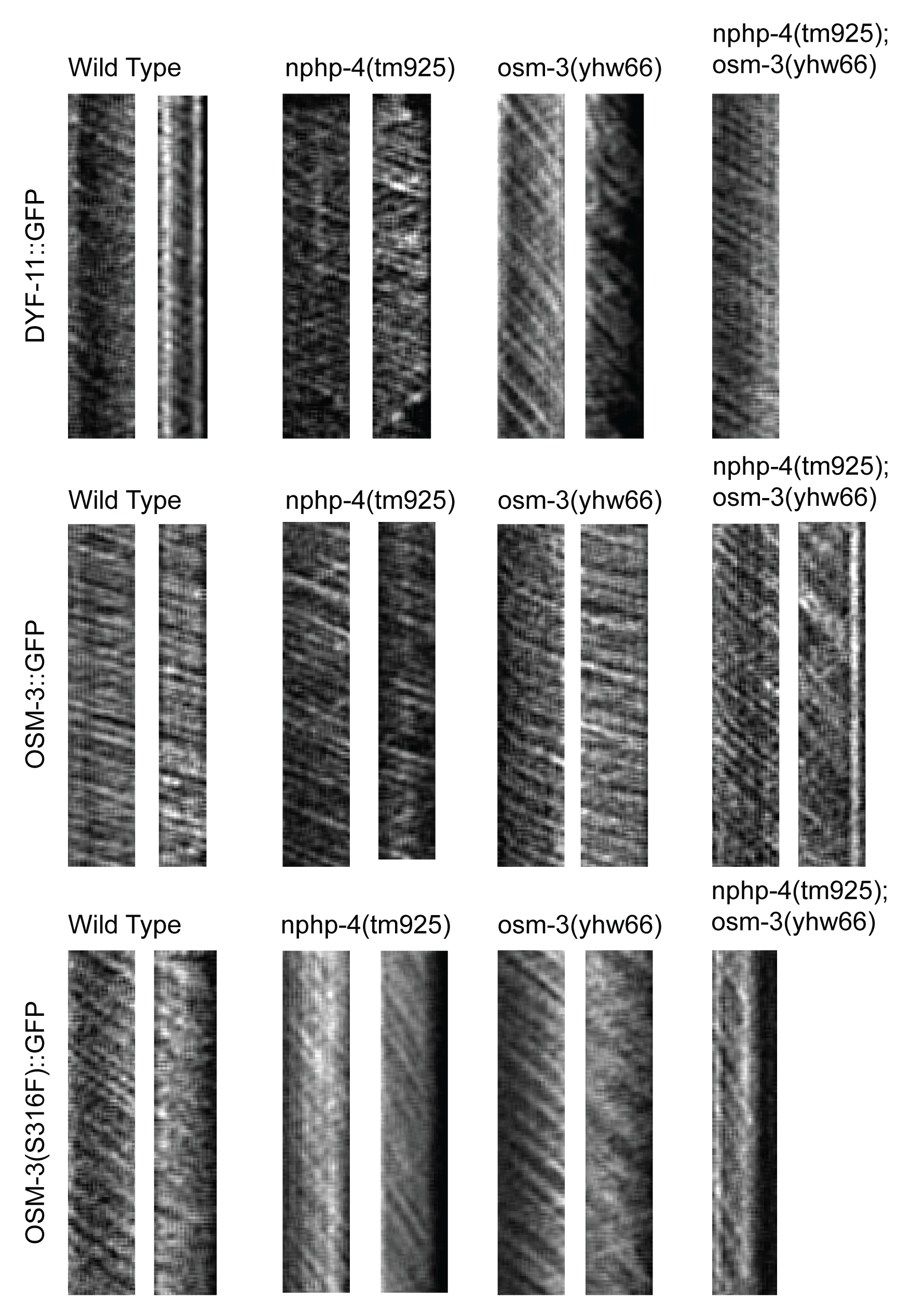

Supplement: S3 Fig — Kymograph images of DYF-11::GFP, OSM-3::GFP, and OSM-3(S316F)::GFP in WT, nphp-4(tm925), osm-3(yhw66), and nphp-4(tm925);osm-3(yhw66). Numerical data for these experiments can be found in Fig 5. MS = Middle segment, DS = Distal segment. (TIF) [file pgen.1005841.s003.tif]
